# Nuclease Footprints in Sperm Project Past and Future Chromatin Regulatory Events

**DOI:** 10.1038/srep25864

**Published:** 2016-05-17

**Authors:** Graham D. Johnson, Meritxell Jodar, Roger Pique-Regi, Stephen A. Krawetz

**Affiliations:** 1 Center for Molecular Medicine and Genetics, Wayne State University School of Medicine, Detroit, MI, 48201, USA; 2 Department of Obstetrics and Gynecology, Wayne State University School of Medicine, Detroit, MI, 48201, USA

## Abstract

Nuclear remodeling to a condensed state is a hallmark of spermatogenesis. This is achieved by replacement of histones with protamines. Regions retaining nucleosomes may be of functional significance. To determine their potential roles, sperm from wild type and transgenic mice harboring a single copy insert of the human protamine cluster were subjected to Micrococcal Nuclease-seq. CENTIPEDE, a hierarchical Bayesian model, was used to identify multiple spatial patterns, "footprints", of MNase-seq reads along the sperm genome. Regions predicted by CENTIPEDE analysis to be bound by a regulatory factor in sperm were correlated with genomic landmarks and higher order chromatin structure datasets to identify potential roles for these factors in regulating either prior or post spermatogenic, i.e., early embryonic events. This approach linked robust endogenous protamine transcription and transgene suppression to its chromatin environment within topologically associated domains. Of the candidate enhancer-bound regulatory proteins, Ctcf, was associated with chromatin domain boundaries in testes and embryonic stem cells. The continuity of Ctcf binding through the murine germline may permit rapid reconstitution of chromatin organization following fertilization. This likely reflects its preparation for early zygotic genome activation and comparatively accelerated preimplantation embryonic development program observed in mouse as compared to human and bull.

Spermatogenesis is characterized by a series of morphological changes resulting in a motile, haploid and highly condensed cell. This is achieved in part through the compaction and restructuring of its nuclear architecture. The haploid expression and progressive deposition of transition proteins (Tnp) and protamines (Prm) within the chromatin fiber displaces the majority of histones from the double helix^1^. The degree to which the histones are replaced varies between species though in mouse it is estimated that between 1–5% of the sperm genome remains histone bound^2^. Once incorporated the protamines compact and silence the genome through the formation of disulfide bridges. Following protamination, the paternal gamete possesses a genomic packaging scheme unlike that of any other cell.

Nuclease mapping in conjunction with high-throughput DNA sequencing has become a powerful tool to rapidly and efficiently survey chromatin landscapes^3^. These approaches can be used to infer chromatin structure in a probabilistic manner based on the relative accessibility of DNA sequences to nuclease cleavage^4^. In sperm, DNAs released following MNase (micrococcal nuclease) digestion are thought to be primarily histone-associated, as evidenced by the discreet banding pattern they produce following electrophoretic resolution^5^. Adapting genome wide MNase mapping approaches to the study of sperm chromatin has highlighted the potential roles that nucleosome-bound DNAs may play in the gamete and following fertilization^6^,^7^,^8^,^9^.

Genomic regulation requires the ordered positioning of DNA within the limited confines of the nucleus. This is accomplished primarily through the folding and looping of chromatin which simultaneously permits interactions between distant genomic regions while reducing the physical volume of the genome. These structural features can be globally mapped through the use of high throughput proximity ligation assays (i.e., Hi-C)^10^,^11^. The preferential chromatin interactions identified by these techniques underlie the basis for dividing the genome into topological associated domains (TADs). These data have become available for various cell types providing a powerful resource for identifying putative cis regulatory partners that may lay beyond a linear DNA segment^12^,^13^.

To understand the cell type specific chromatin packaging strategy employed within the male gamete, mouse sperm were nuclease digested and the concomitant released nucleosome-associated DNAs subjected to high throughput sequencing. The susceptibility of the spermatozoon to enzymatic dissection was compared in sperm from wild type mice and a homozygous transgenic mouse model harboring a single copy insert of the human protamine locus. This 40 kb sequence was stable over many generations and did not alter spermatogenesis or impact fertility^14^. Transcriptomic and proteomic analysis established that their benign phenotype reflected decreased transcriptional activity of the suite of human transgenes as compared to the endogenous mouse locus. To understand its suppression in sperm, a nuclease footprinting approach was undertaken. Regions predicted to be bound by a regulatory factor in mature sperm were correlated with genomic landmarks and higher order chromatin structure datasets to identify potential roles for these factors in regulating either prior or post spermatogenic, i.e., early embryonic, events. This analysis identified a series of candidate enhancer-bound regulatory proteins that as mediated by Ctcf-DNA looping are expected to contribute to the robust expression of the endogenous protamines. Genome wide analysis of Ctcf binding suggested potential functions for this factor in the mouse gamete and embryo. Interspecies comparison of nuclease footprints failed to identify the presence of Ctcf in either human or bull sperm strongly suggesting its role(s) following fertilization are likely species specific.

## Results

### Nuclease sensitivity in wild type and transgenic mouse spermatozoa

**Figure 1:**
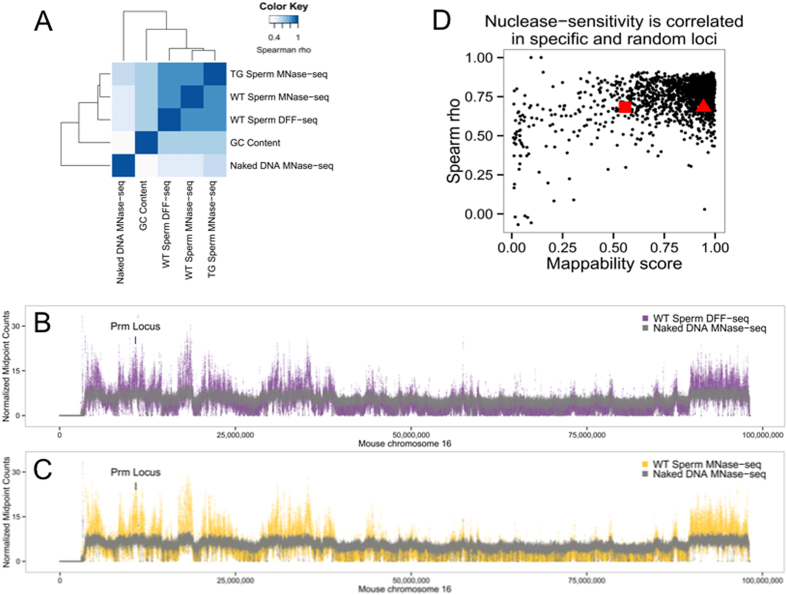
Mouse sperm chromatin nuclease-sensitivity is correlated across all samples. (**A**) Hierarchical clustering of pairwise spearman rho correlation coefficients for genome-wide nuclease-sensitivity data from *in situ* digested sperm chromatin binned into 500 base pair windows demonstrated consistent nucleosome coverage regardless of nuclease choice or genotype, relative to naked DNA controls. GC content represents the average percentage of GC dinucleotides in the window. (**B,C**) Sperm chromatin exhibits similar patterns of nuclease-sensitivity regardless of digestion with MNase of DFF (purple and orange, respectively, relative to naked DNAs (gray). Coverage of chromosome 16 is presented as normalized paired-end sequencing midpoint counts in 500 base pair windows. (**D**) Sperm MNase-seq coverage of the endogenous mouse protamine locus in (red triangle; wild type and transgenic sperm), the sequences flanking the transgene insertion site (red square; wild type and transgenic sperm), and 2,000 randomly selected loci were well correlated (y axis) and only marginally influenced by (x axis) mappability. The midpoints of paired-end sequencing fragments were binned into 50 base pair windows along 20 kb regions and correlation coefficient calculated using a 5 window running average.

Nucleosome-associated DNAs were released from wild type and transgenic mouse sperm with either Micrococcal Nuclease (MNase) or DNA fragmentation factor (DFF)^15^,^16^,^17^. Use of the latter nuclease provided a unique complimentary approach to probe sperm chromatin structure and served as an additional control for MNase cleavage bias^18^,^19^. Unlike MNase that has been proposed to cleave DNA along the dyad axis, the nuclease activity of DFF is restricted to nucleosomal linker regions due to the large size and steric positioning of the dimerized enzyme. Genome-wide nuclease sensitivity was well correlated amongst sperm samples (ρ ~ 0.89–0.91) and distinct from that observed following digestion of purified DNAs (Fig. 1). Nucleosome retention varied across sperm chromosomes highlighting the presence of broad regions of heightened nuclease sensitivity that could not be explained by GC content (Fig. 1A). Within mouse chromosome 16 the endogenous protamine domain resides within a region of elevated nuclease sensitivity (Fig. 1B,C). In comparison, transgenic sperm did not exhibit altered genome wide nuclease sensitivity despite the integration of an additional protamine locus. To assess the impact of this inserted human locus, MNase-seq coverage of specific regions of the mouse genome were correlated. Applying this approach to 20 kb regions centered on the endogenous mouse protamine locus, sequences flanking the site of transgene integration (chr19:39,397,384-39,397,385)^14^, or on randomly selected regions of equal length demonstrated that nuclease sensitivity within these regions was similar (ρ ~ 0.68) across all samples (Fig. 1D).

In contrast to a prior study that interrogated crosslinked mouse sperm chromatin, nucleosome coverage was not elevated within gene deserts (Supplemental Figure 1A)^20^. Sperm nucleosomes were enriched within transcription start sites, relative to control DNAs, as has previously been reported for analyzes of native human and mouse sperm chromatin (Supplemental Figure 1B)^6^,^8^. In the current study, mono- and polynucleosomal associated sperm DNAs were released following digestion with MNase (Supplemental Figure 1C) suggesting that the results reported herein do not reflect “over-digestion” of the paternal chromatin.

**Figure 2:**
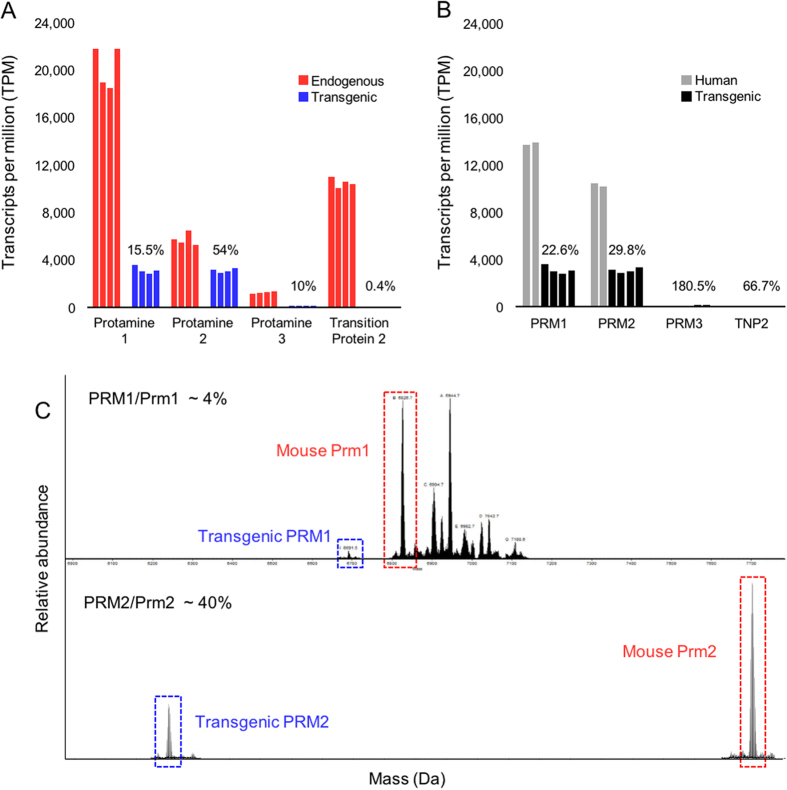
Expression of the human transgenes in mouse testis was suppressed. (**A,B**) RNA-seq analysis of transgenic mouse (n = 4, (**A**)) and human (n = 2 (**B**)) GSE69434) total testes RNAs demonstrated that transcription of the integrated human protamine genes was reduced relative to that observed from both the endogenous loci. Expression values are presented as transcripts per million (TPM). TPM ratios for each comparison are presented above the transgenes. (**C**) Acid extracted transgenic mouse sperm chromatin proteins were analyzed by mass spectrometry. The amount of transgenic (human) protamines (blue) incorporated into mouse chromatin was reduced relative to the endogenous protamine proteins (red). Peptides exhibiting mass to charge ratios exceeding that observed for mouse Prm1 may correspond to post-translational modifications such as phosphorylation^79^.

Testis RNA and sperm protein levels were analyzed to determine if the absence of altered chromatin structure in transgenic sperm was due to transcriptional/translational regulation or impaired incorporation of the transgenic proteins during chromatin condensation. RNA-seq analysis of total transgenic testis RNAs demonstrated that the transgenes were transcribed but exhibited reduced expression relative to their orthologs and analogs in mouse and human testis (GSE69434), respectively (Fig. 2A,B). The average ratio of the transgenic protamines (TG PRM1/TG PRM2 ~ 1.02) differed from that observed in human (Hs PRM1/Hs PRM2 ~ 1.34) and wild type mouse (Mm Prm1/Mm Prm2 ~ 3.57) testes. Likewise, transgenic PRM3 and transgenic TNP2 RNA levels were similarly altered relative to wild-type and human testis RNA levels. Analysis of acid extracted transgenic sperm chromatin proteins demonstrated that incorporation of the transgenic proteins was reduced relative to that observed for the endogenous proteins and complimented the relative abundance of the corresponding RNAs in testis (Fig. 2C).

### Chromatin structure of the human and transgenic protamine locus

**Figure 3:**
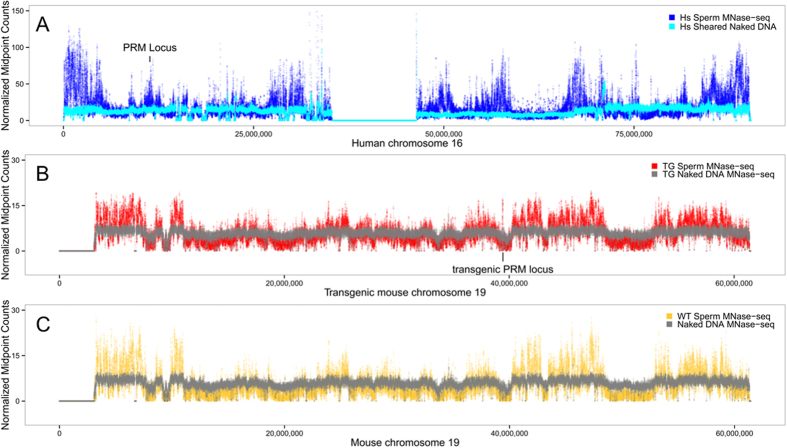
The human protamine sequences in sperm are packaged within nuclease-sensitive chromatin. (**A,B**) The human protamine locus exhibits elevated nuclease sensitivity in mouse and man but is associated with regional variations in MNase-seq coverage. Normalized paired-end sequencing coverage of human and transgenic sperm MNase-seq libraries are presented relative to sheered or digested controls, respectively. (**A**) The endogenous human protamine locus resides within a large region of elevated nuclease sensitivity. (**B**) In contrast while the transgenic domain is locally enriched in MNase-seq coverage it resides within a nuclease insensitive region of mouse chromosome 19. (**C**) The presence of the transgenes did not impact the chromatin structure of the neighboring sequences as this region is also nuclease insensitive in wild type mouse sperm.

To identify potential causes of transgene suppression nuclease sensitivity within the transgenic and human protamine loci were compared. MNase digestion of transgenic mouse and human sperm chromatin demonstrated that the human protamine locus exhibited elevated nuclease sensitivity in either context (Fig. 3A,B). However, in contrast to the endogenous protamine loci in mouse (Fig. 1) and human sperm the nuclease sensitive conformation of the transgenic protamine sequence was flanked by nuclease-insensitive DNA (Fig. 3B). In human sperm the protamine gene cluster resides within an extended region of elevated nuclease sensitivity relative to control DNAs, reminiscent of that observed for the orthologous sequences in mouse sperm (Fig. 3A).

**Figure 4:**
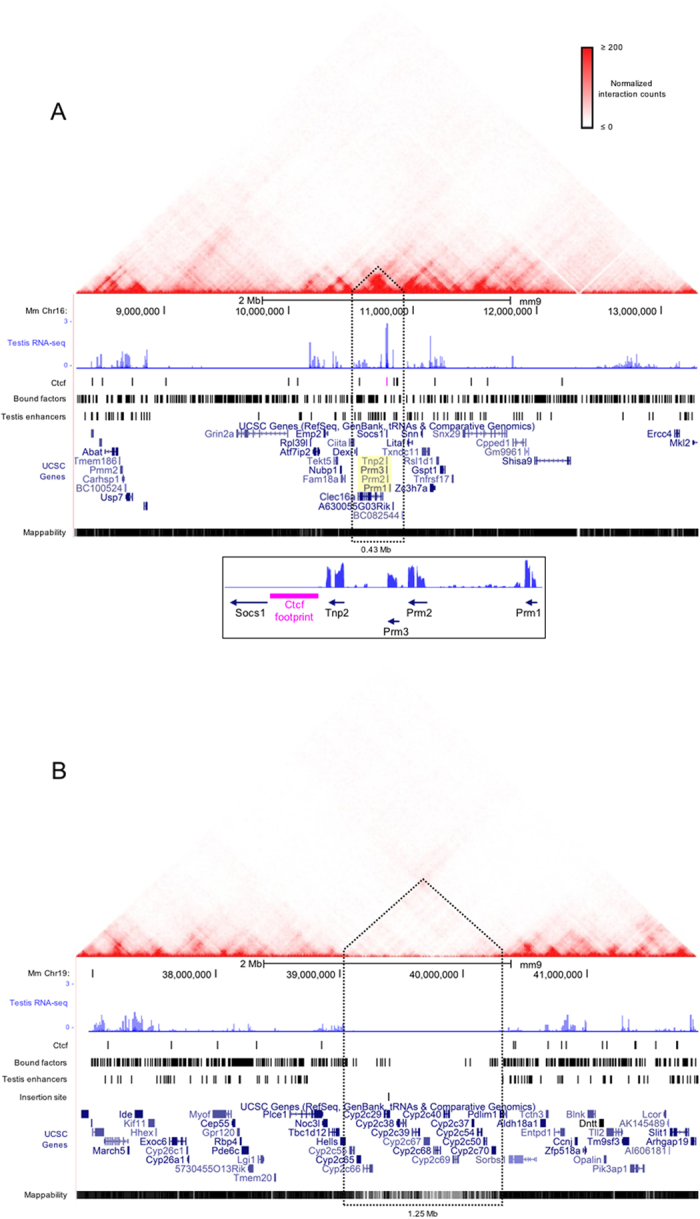
The chromatin domains harboring the abundantly expressed endogenous protamine locus and the suppressed transgenes exhibit different regulatory features. The relative positioning of cis regulatory elements and testis RNA-seq coverage along 5 Mb regions centered on (**A**) the mouse protamine locus (inset) or (**B**) the transgene insertion site (chr19:39,397,384-39,397,385), is presented within the context of intra-chromosomal contact data from Hi-C analyses of CH-12 cells^12^. Mean coverage of uniquely aligned sequencing reads from total transgenic testes RNAs (n = 4) aligned to the wild type mouse genome are presented as log_10_ normalized values. Nuclease footprints corresponding to Ctcf and other factors bound in mature mouse sperm are displayed as separate tracks. The Ctcf footprint positioned between Socs1 and Tnp2 is highlighted in pink. The protamine gene cluster and ~2 kb Ctcf footprint is displayed in a separate lower panel. Predicted testis single nucleotide enhancer peaks were provided by the mouse ENCODE project^36^. The locations of the endogenous protamines are highlighted in yellow. The site of transgene insertion within mouse chromosomes 19 is marked on a separate track. Peaks of Hi-C interaction frequencies containing the loci of interest are demarcated by dashed lines and are considered chromatin subdomains. (Inset) The endogenous protamine gene cluster is displayed along with the Ctcf footprint (pink) identified between Socs1 and Tnp2.

Candidate chromatin and genomic features that may contribute to haploid PRM transcription and nuclease sensitivity in sperm were identified within 5 Mb regions centered on the protamine loci (Fig. 4). Analysis of available Hi-C chromatin interaction datasets showed that the endogenous mouse and human gene clusters (Fig. 4A and Supplemental Figure 2) lay within approximately 0.5 Mb domains enriched in intrachromosomal contacts. These domains are largely invariant in all cell types examined (Supplemental Figure 3)^11^,^21^. The 5 Mb region encompassing the mouse protamine gene cluster contains 100 ENCODE predicted testis enhancers of which 18 are within the subdomain housing the protamine genes (Fig. 4A). In contrast to the chromatin domains harboring the endogenous PRM gene clusters, the transgenes integrated into a comparatively large, repeat dense region exhibiting relatively fewer intrachromosomal interaction events and no predicted testis enhancers (Fig. 4B).

The chromatin domains harboring the endogenous and transgenic protamine loci exhibited varied amounts of intrachromosomal contacts. Domain structure is known to be demarcated by Ctcf binding which is also responsible for mediating the DNA looping events. Analysis of round spermatid Ctcf ChIP-seq data^22^ identified 102 Ctcf peaks within the 5 Mb region and 17 Ctcf peaks within subdomain harboring the endogenous mouse protamine domain (Fig. 4A). Several of the Ctcf peaks are located immediately upstream of Tnp2 and intersect a previously identified nuclear matrix attachment site^23^,^24^. This sequence is conserved in humans and mutations within this region are correlated with infertility in men^25^ while the absence of this sequence in prior transgenic models of the human protamine locus subjects the transgenes to position effects^26^. In the absence of mature sperm ChIP-seq data, genome wide Ctcf occupancy was inferred from MNase-seq data using the CENTIPEDE^4^ algorithm. CENTIPEDE employs a negative-multinomial distribution to model the spatial pattern of fragment midpoints around instances of transcription factor binding motifs. Ctcf binding to the motif lying between Socs1 and Tnp2 that is bound in round spermatids during the window of Prm expression^22^, was predicted to remain bound in mature sperm (Fig. 4A). This region of the endogenous protamine domain was also predicted to be bound in transgenic sperm. Similarly, occupancy of the syntenic CTCF motif upstream of TNP2 within the nuclease sensitive transgenic human protamine locus was inferred by CENTIPEDE. This site must be bound prior to nuclear condensation suggesting that its utilization by Ctcf may be functionally equivalent as that observed for the site positioned within the endogenous mouse protamine locus.

### Mouse spermatozoa harbor bound chromatin factors

Round spermatid ChIP-seq and sperm MNase-seq data were used to infer the binding status of Ctcf. In mature sperm this factor is predicted to remain bound to conserved motifs within the endogenous and transgenic protamine loci. In wild type mice this association was verified in the preceding cell type and presumed to be similarly bound within the transgenic sequence. This suggested that motifs contained within the transgenic protamine locus could be accessed and bound by their cognate factors. Therefore, suppression of the transgenes was likely not to due blocked protein binding within the integrated locus. However, removed from its endogenous chromatin domain the transgenic protamines would be reliant upon factor(s) binding to neighboring DNA elements to contribute to locus control.

**Figure 5:**
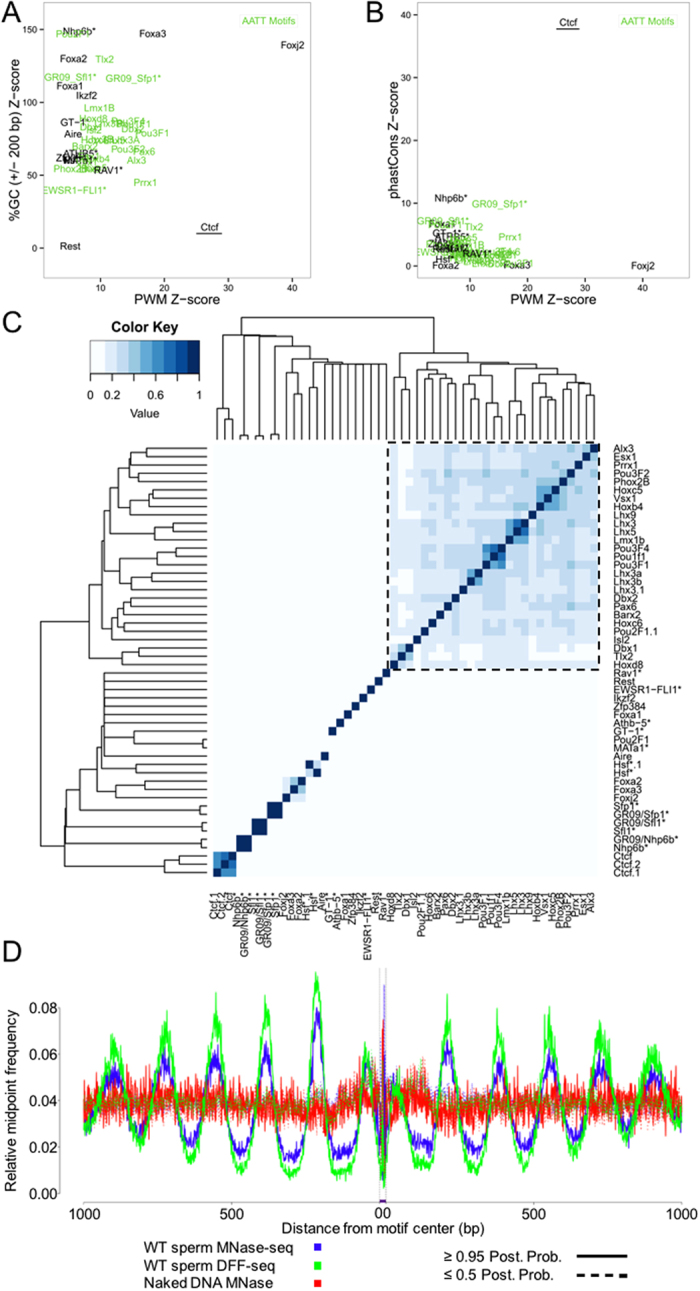
Nuclease footprinting establishes the presence of bound regulatory factors in mouse sperm chromatin. Pooled nuclease-seq (MNase and DFF) data sets and PWM values were used to infer the occupancy status of regulatory factor binding sites in mouse sperm. The potential predictive value of additional variables to factor binding predictions was assessed with a generalized linear model. PWM Z-scores were compared to those corresponding to either (**A**) GC content (%GC ± 200 motif) or (**B**) conservation within the motif (phastCons). Alternative variable Z-scores are plotted against PWM Z-score for each of the fifty-two motifs for which PWM was predictive of binding (Z-scores > 5). (**C**) Similarity (Jaccard index) heat map of genomic coordinates for factors bound in sperm. Homeobox factors (right hand cluster, dashed box) bind a highly similar motif and consequently cluster together. Factors in this cluster are labelled in green in panels A and B. Asterisks denote motifs identified in other species with no known mouse homolog. (**D**) Bound motifs in sperm are flanked by poly-nucleosome arrays. Ctcf footprints from Wild Type sperm digested with either MNase or DFF (blue and green, respectively) are revealed by plotting sequencing fragment midpoints aligned within the regions flanking bound motifs (solid line, posterior probability ≥ 0.95). Sequencing coverage proximal to bound sites is correlated with periodic increases in midpoint frequency approximately every 160 bp reflecting an increase in protected nucleosome-bound DNA relative to digested linker regions. In contrast, unoccupied motifs in mouse sperm (dashed line, posterior probability ≤ 0. 5) and naked control DNAs (red) do not exhibit variable levels of nuclease susceptibility.

To determine whether the mouse spermatozoal chromatin landscape contains factors of potential regulatory importance in addition to Ctcf, sites corresponding to known position weight matrices (PWMs)^27^,^28^ were identified throughout the genome and their occupancy deduced from the sperm nuclease-seq datasets with CENTIPEDE^4^. Regardless of genotype (TG or WT) or nuclease selection (MNase vs. DFF) posterior probabilities of binding were well correlated for all factors in which motif PWM values were predictive of the CENTIPEDE footprint (Z-score ≥ 5; Supplemental Figure 4). The sperm datasets were pooled, removing alignments to the integrated transgenic DNA and reanalyzed identifying 46 chromatin factors (52 motifs) predicted to be bound in mature sperm (Fig. 5). In addition to the PWM value, the influence of GC content (±200 bp motif) and local sequence conservation on occupancy were estimated using a generalized linear model (Materials and Methods equation 1). The CENTIPEDE Ctcf model was a clear outlier exhibiting elevated conservation and PWM Z-scores and was only modestly influenced by the sequence context neighboring the factor motif(s) (Fig. 5A,B).

Hierarchical clustering of bound motifs according to their genomic distributions (Jaccard similarity index) identified a subset of overlapping motifs utilized by the homeobox domain protein family (n = 27; Fig. 5C, dashed box; Supplemental Figure 5, purple box). The proteins in this cluster possess highly similar PWMs (Supplemental Figure 6) but exhibit a broad range of RNA levels in testis (Supplemental Data 1). Within this subset of proposed sperm chromatin factors Pax6 (Paired box 6) and Esx1 (Extra-embryonic tissue-spermatogenesis-homeobox gene 1) have been previously observed in testis^29^. However, Pax6 was not detected in mature sperm and its relative RNA level in testis is 3% that of Esx1. In contrast, of all the homeobox family members predicted to be bound in sperm, Esx1 RNA levels were the 2^nd^ most abundant of these factors in testis (Supplemental Table 1; Supplemental Data 1). The Esx1 protein has been localized to late spermiogenic cell types including the mature gamete^30^.

Approximately 95–99% of the histones have undergone replacement by protamines following spermatogenic nuclear remodeling in mouse, yet MNase digestion of sperm chromatin reveals a similar relationship between several chromatin binding proteins and nucleosome periodicity in the histone-depleted gamete. Recapitulating observations from somatic cells^31^, this nucleosome periodicity extends for approximately 1.5 kb in sperm (Fig. 5D; solid green and blue lines). In contrast, aggregate nucleosome coverage is indistinguishable from control DNAs in the absence of a binding event (Fig. 5D; dashed green and blue lines vs. solid red lines). Prioritizing motifs with a corresponding testis expressed factor and a nuclease footprint indicative of flanking nucleosome periodicity identified 17 sperm chromatin bound factors (Supplemental Table 1; Supplemental Data 2, 3). Two Pou2f1 (POU domain, class 2, transcription factor 1) motifs were partitioned into separate groups after hierarchical clustering (Supplemental Figure 5). A prior study identified this factor in mature mouse sperm and correlated its motif with nuclease sensitive DNAs^32^. In the same way as Ctcf, Pax6, Esx1, and Pou2f1, two members of the winged helix transcription factor family are expected to be bound in sperm and have previously been identified in the male germline. Similar to Pax6, both Foxj2 (Forkhead box J2) and Foxa3 (Forkhead box A3) are expressed in round spermatids and in other testicular cell types, but have yet to be identified in the mature gamete suggesting that if bound in sperm the factors are not abundant^33^,^34^,^35^. The remaining nuclease footprinted nucleosome associated factors await confirmation in mouse sperm.

### Sperm chromatin bound factors are enriched within regulatory regions

To infer likely prior and future functions of the bound motifs observed in mature sperm chromatin, their occupancy in known regulatory regions and promoters were compared. Analysis of ENCODE testis ChIP-seq datasets highlighted associations between nuclease footprints and active chromatin features^36^. Regions of Ctcf, Foxj2 and Rest (RE1-silencing transcription factor) binding in sperm were significantly associated with peaks of active histone modifications including H3K4me3 and H3K27ac (P < 3.7 × 10^−16^ – 6.2 × 10^−286^). Rest, Foxj2, and Pou2f1 sites in sperm were also significantly enriched within promoters of genes expressed in the male germline (P < 8.2 × 10^−6^–1.9 × 10^−79^), residing within regions marked by active histone modifications in testis. Motifs bound by Foxj2 and Pou2f1 were found within 4,386 testis promoters suggesting that these transcription factors are important regulators of spermatogenic transcription. A similar analysis of all RefSeq gene promoters demonstrated that only Rest exhibited a significant enrichment within this broad set of regions (P < 2.5 × 10^−171^). Ctcf sperm footprints were not enriched in the above promoter sets but were significantly associated with predicted testis enhancers reflecting the role of this factor in regulating chromatin interactions (P < 3.6 × 10^−224^). Bound motifs corresponding to Foxj2 and Rest were also significantly associated with testis enhancers though to a lesser degree (P < 2.7 × 10^−3^ and P < 0.014, respectively).

**Figure 6:**
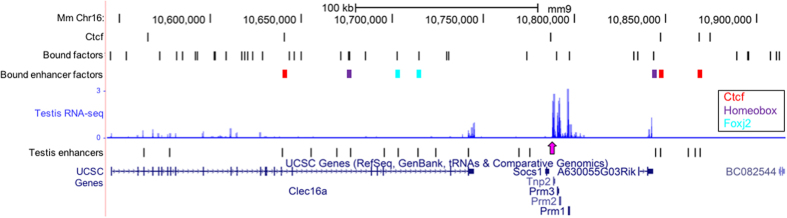
Footprint analysis of endogenous protamine locus. To identify potential regulators of prior endogenous protamine transcription the CENTIPEDE footprint analysis was relaxed to test the occupancy status of additional motifs found within the 5 Mb region centered on the mouse protamine locus. Footprints (2 kb CENTIPEDE window; see Methods) overlapping testes enhancers are colored according to their name. Average log_10_ normalized total testis RNA-seq coverage is depicted in blue. Pink arrow designates the Ctcf site bound upstream of Tnp2. Predicted testis enhancer peaks were provided by the mouse ENCODE project (Methods)^36^. Additional footprints identified in sperm that do not intersect a testis enhancer are presented in a separate track (motif length, ~20 bp).

To identify candidate regulators of protamine transcription the distribution of sperm nuclease footprints was determined within the 5 Mb search regions (Fig. 4) housing the protamine gene clusters. A similar number of footprints were observed within these extended regions of interest on chromosomes 19 and 16 (n = 650 and 598, respectively) as well as within the chromatin domains^23^,^37^,^38^ harboring the transgenes and mouse protamines (n = 50 and 43, respectively). However, the relative density of footprints was greater proximal to the endogenous gene cluster reflecting the reduced size of the domain containing these sequences. Ctcf exhibited a limited presence in the larger domain harboring the integrated transgenic sequences. This region contained only seven spermatid Ctcf ChIP-seq peaks and no corresponding Ctcf footprints in sperm (Fig. 4B), with the exception of the single SOCS1-TNP2 Ctcf footprint present within the human transgenic construct. In contrast the endogenous Prm domain was relatively enriched in Ctcf, containing 17 ChIP-seq peaks and 3 sperm Ctcf footprints (Fig. 4A). To detect factors that may have contributed to regulating the expression of the protamine locus through binding testis enhancer elements, the initial stringent footprinting analysis was repeated, relaxing parameters for sites identified in either 5 Mb search region (Methods). This expanded analysis identified six footprints overlapping predicted enhancers within the endogenous Prm domain. Additional occupied sites were also localized within the protamine gene cluster (Fig. 6). Factors predicted to be bound to enhancer elements in sperm (Table 1) are expected to have been inherited from prior cell types as observed for Ctcf in round spermatids and the mature gamete.

### Sperm chromatin bound factors are enriched near sites of embryonic transcription

The group of homeobox domain motifs identified by hierarchical clustering did not exhibit a significant association with testis promoters or regulatory regions. Rather this group of related factors, including Hoxd8 (Homeobox D8), Tlx2 (T cell leukemia, homeobox 2), and Lhx5 (LIM homeobox protein 5), were significantly enriched upstream of ribosomal RNA sequences (P < 2.7 × 10^−3^–5.5 × 10^−8^). Transcription of these sequences has recently been demonstrated as necessary for zygotic maturation^39^. Foxa3 and Foxj2 sperm footprints also exhibited a significant association with these regions (P < 6.4 × 10^−6^ and P < 2.5 × 10^−4^, respectively).

**Figure 7:**
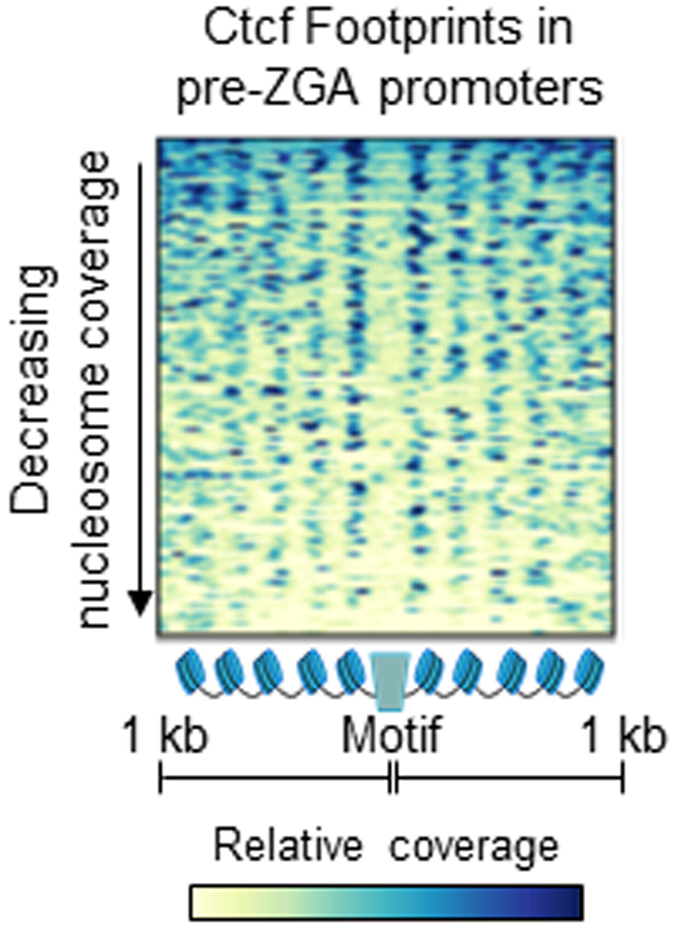
Mouse sperm Ctcf sites are flanked by nucleosomes enriched within the promoters of genes expressed in the zygote. Pooled sperm nucleosome coverage is shown within 2 kb regions (n = 106) centered on Ctcf motifs predicted to be occupied in sperm and intersect promoters of differentially expressed genes in pronuclear embryos relative to oocytes (n = 106)^59^. The enrichment of Ctcf footprints within this set of promoters was statistically significant (P < 2.8 × 10^−284^, Fisher exact test). Elevated nucleosome sequencing coverage is in blue and regions are ordered by decreasing total nucleosome coverage.

Sperm derived nucleosomes may also contribute to the establishment of embryonic chromatin in other regions of the genome. Dysregulation of spermatogenic polyADP-ribose metabolism alters histone positioning within the gamete and is correlated with perturbed expression of the olfactory receptor genes in the 2-cell embryos sired by treated males^40^. Homeobox domain footprints identified in sperm were significantly enriched in the promoters of this gene family (merged homeobox sites, P < 1.7 × 10^−38^). In total the merged set of bound homeobox domain motifs overlapped 31% of all olfactory receptor gene promoters (354/1130). The alternative Pou2f1 motif was also significantly enriched within these regions (n = 151, P < 3.5 × 10^−96^). Similarly, a set of Ctcf nuclease footprints was enriched within the promoters of genes differentially expressed in mouse embryo pronuclei relative to oocytes (n = 106, P < 2.8 × 10^−284^; Fig. 7). These footprints are accompanied by well positioned arrays of polynucleosomes that overlap the promoter sequences thereby imparting a preferentially accessible structure necessary for early utilization by the embryo.

### Characterization of Ctcf in mouse sperm

Nucleosome coverage across the endogenous and transgenic loci suggested a potential role for Ctcf in coordinating expression of these sequences. In both somatic cells and sperm, the binding of Ctcf in addition to establishing chromatin domains, locally results in well positioned arrays of polynucleosomes (Fig. 5D)^41^,^42^. In the male gamete, these nucleosome arrays contained both canonical and replication-independent histones (Supplemental Figure 7). Ctcf footprints were also observed in modified H3K27me3 but not H3K4me3 sperm datasets^8^ likely reflecting the varied distributions (broad and diffuse vs. narrow and dense, respectively) of the opposing histone modifications^36^. However, the role that Ctcf serves within the static sperm nucleus remains unclear, though any proposed functions must bookend sperm maturation and fertilization due to chromatin condensation.

**Figure 8:**
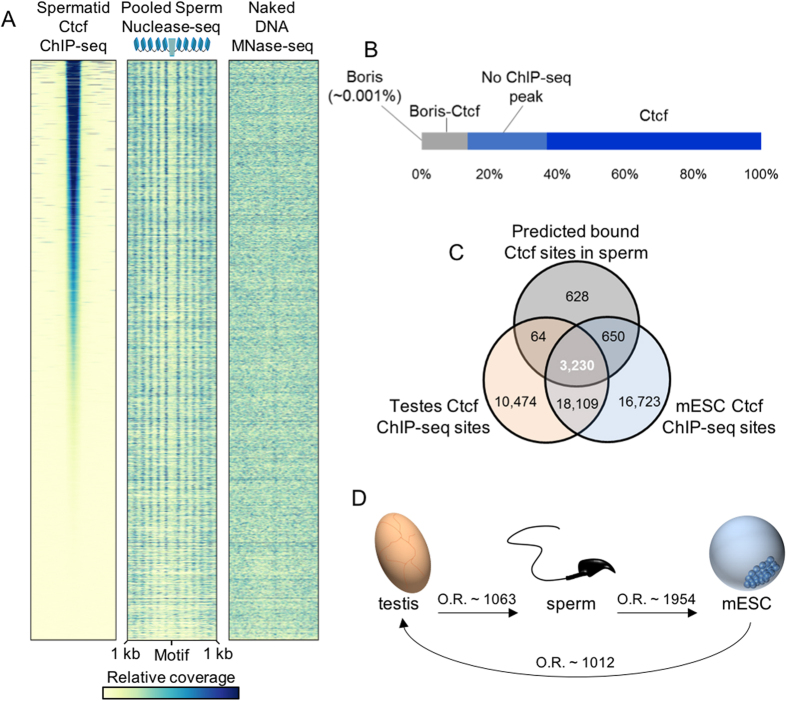
Ctcf remains bound in mature sperm following chromatin remodeling in mouse. (**A**) Heatmaps of relative sequencing coverage in 2 kb windows centered on the complete set of 7,967 Ctcf sites bound in mature mouse sperm (Methods). Data are presented for ChIP-seq against Ctcf in round spermatids^22^, sperm nuclease-seq, and digested naked DNA libraries (left, center, and right, respectively). Sites are ranked according to decreasing ChIP-seq coverage. (**B**) The majority of the Ctcf sites occupied in sperm (n = 7,967) are occupied solely by Ctcf in round spermatids (n = 5,034, 63.2%). A subset of sperm Ctcf sites correspond to co-occupied Boris-Ctcf peaks in round spermatids (n = 1,075, 13.5%; Supplemental Figure 8B) or lack a corresponding ChIP-seq peak in the prior cell type (n = 1,857, 23.3%). In the latter case this was due to peaks not achieving significance (Supplemental Figure 8A). (**C,D**) The majority of Ctcf sites (Motif M01200) bound in sperm are observed in mouse ENCODE testis and mESC ChIP-seq datasets. Odds ratios (O.R.) from Fisher exact tests are presented between cell types comparisons.

Ctcf footprints identified in sperm significantly overlapped ChIP-seq peaks associated with this factor in round spermatids (P < 2.2e-16, Fisher exact test; Fig. 8A)^22^. Greater than 86% of the Ctcf motifs predicted to be occupied in the male gamete (n = 5009/5797) correspond to a binding event in the earlier cell type (Fig. 8B). Relaxing the minimum PWM value (PWM value ≥ 13; Methods) used to identify Ctcf binding sites in sperm returned 2,170 additional footprints of which approximately 76.5% overlapped a spermatid ChIP-seq peak (n = 6,109/7,967). Ctcf motifs predicted to be occupied in sperm which lacked a corresponding ChIP-seq peak reflect sites that failed to reach significance in spermatids and not the presence of exclusive binding within the gamete (Supplemental Figure 8A). A minor subset of sperm Ctcf footprints (~13.5%, Fig. 8B) coincide with regions bound by both Ctcf and the highly related protein Boris (Brother of Regulator of Imprinted Sites; Supplemental Figure 8B). Contrary to a recent report^22^, several independent observations suggest that in mature mouse sperm, Ctcf is primarily associated with nucleosomes and not Boris. The latter factor has been localized to pre-meiotic spermatogenic cells^43^ and appears to be gradually depleted following meiosis as demonstrated by a strong reduction in ChIP-seq peaks in round spermatids, relative to that observed for Ctcf (n = 5,393_Boris_ and 42,493_Ctcf_).

ChIP-seq analysis of Ctcf binding in round spermatids and CENTIPEDE footprinting in mature sperm suggest that of the 42,493 Ctcf peaks identified in the prior cell type approximately 82% are evicted during the histone-protamine transition. Motifs that remain occupied in mouse sperm are significantly enriched within Ctcf ChIP-seq peaks from testis and embryonic stem cells, although the sites occupied by this factor in sperm are relatively few in number (P < 0.0001, odds ratio ~1063; Fig. 8C,D). Ctcf footprints detected in mature sperm were log-normally distributed along the chromosomes, exhibiting a median distance between sites of approximately 230.5 kb (S.D. ~ 1 Mb; Supplemental Figure 9). Occupied mouse sperm Ctcf motifs (posterior probability > 0.95) were also significantly enriched within the boundaries of embryonic stem cell (ESC) TADs (P < 4.3 × 10^−232^; p < 1.0 × 10^−4^, Empirical p-value) to a greater degree than unbound sites (posterior probability > 0.5; P < 1.8 × 10^−20^; P < 4.5 × 10^−2^, Empirical p-value).

### Ctcf is absent in man and bull

**Figure 9:**
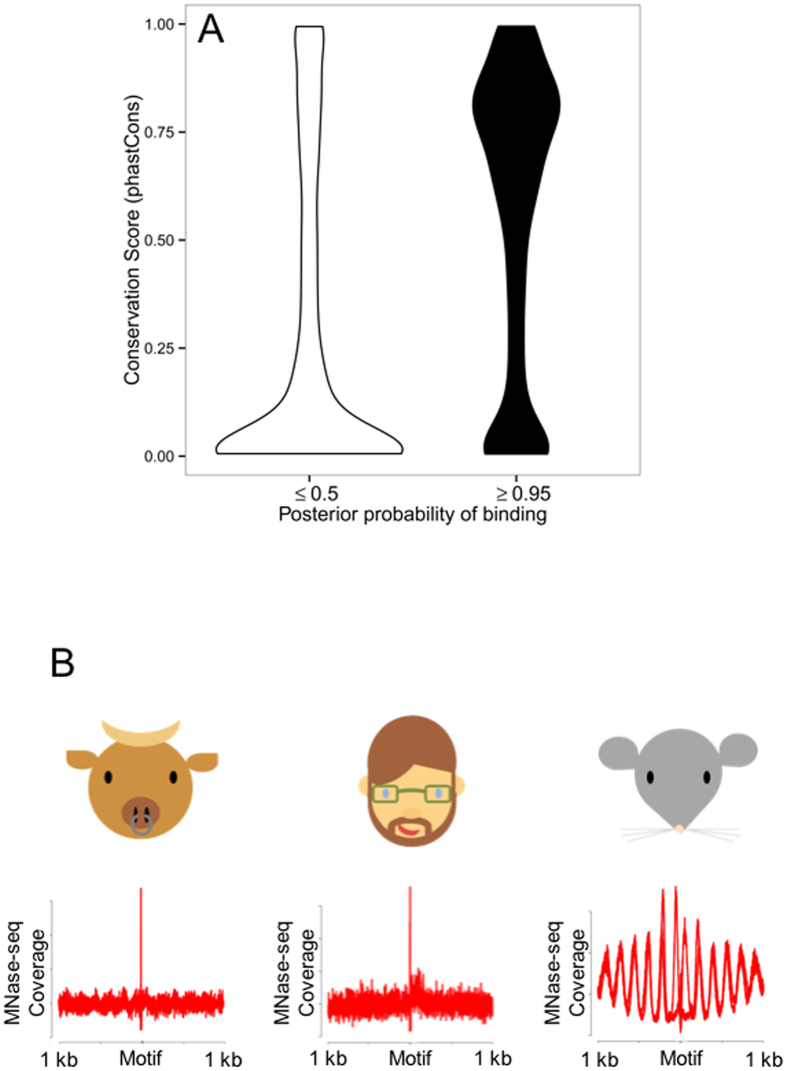
Ctcf footprints in mouse sperm are highly conserved but are not occupied in men and bulls. (**A**) CENTIPEDE Ctcf motifs bound in mouse sperm (posterior probability ≥ 0.95, black) exhibit greater sequence conservation (phastCons; 60 placental mammalian genomes) than unbound sites (posterior probability ≤ 0.5, white). (**B**) Ctcf footprints are not resolved following MNase digestion of human or bull sperm chromatin. CENTIPEDE footprint plots depicting the aggregate distribution of MNase-seq coverage flanking Ctcf motifs are shown for bull, human and mouse sperm. Note, that in contrast to human and bovine, only mouse shows nucleosome periodicity.

Ctcf motifs predicted to be bound in murine spermatozoa (posterior probability > 0.95) exhibited a greater degree of conservation than sites lacking a nuclease footprint (posterior probability > 0.5; P < 2.2 × 10−16, Mann–Whitney U-test; Fig. 9A). This suggested that the retention of this chromatin insulator in sperm may be a conserved facet of mammalian paternal gametes. Prior studies have correlated sequences containing CTCF binding motifs with nucleosome-associated DNAs in human sperm^7^ and transcripts encoding this factor are abundant in human testis (Supplemental Data 1). Subjecting human and bull^44^ sperm mononucleosome MNase-seq libraries to the CENTIPEDE footprinting revealed that Ctcf is not bound in mature sperm of either species (Fig. 9B). The availability of previously published human sperm datasets from two independent laboratories, in addition to the human sperm MNase-seq data reported herein, indicated that this finding was sample independent and observed regardless of the experimental protocol employed^9^,^44^. Further, exhaustive analysis of available PWMs indicated that well phased polynucleosomal arrays are not associated with any known motifs in human sperm (Supplemental Figure 10). The Ctcf footprint within the transgenic sperm human protamine domain directly contrasted that observed in human sperm further supporting the conclusion that chromatin packaging in mammalian sperm is not reliant on a single feature such as sequence-context but driven by another, or the combination of several features.

## Discussion

To dissect chromatin packaging in the mature male gamete, nucleosome bound DNAs were enzymatically released from wild type and transgenic mouse sperm. The susceptibility of the transgenic sperm chromatin to nuclease attack was not perturbed despite the presence of an additional protamine locus in these cells (Fig. 1). The integrated human protamine gene cluster also exhibited elevated levels of nuclease digestion relative to controls. This is similar to the nuclease sensitivity exhibited by the endogenous mouse and human protamine loci (Figs 1 and 2A), which has been shown to lie within a DNase I-sensitive structure established during meiosis then persisting throughout^23^,^45^. In humans and mice, the chromatin conformation of this region of chromosome 16, assumed prior to nuclear condensation, may reflect the generalized nuclease sensitivity of a larger domain which persists within the mature male gamete^37^,^38^,^45^.

Analysis of total transgenic and human testis RNAs demonstrated the failure of the transgenes to achieve the levels of expression of either the endogenous human or mouse gene clusters (Fig. 2A,B). This was apparent in the reduction of the transgenic PRM RNAs to 70–77% relative to that observed in man. Indeed, all transgenic RNAs, except PRM3, were significantly reduced relative to either of their endogenous counterparts in mice or man. Transgenic PRM3 was nearly double that observed in human testis, however this transcript is present at relatively reduced levels (Fig. 2B). Proteomic analysis of transgenic sperm chromatin demonstrated that the relative amount of protamine protein utilized by the gamete directly corresponded to RNA availability (Fig. 2C). These results are in accord with the view that the similarity in nuclease sensitivity observed in wild type and transgenic mouse sperm was likely due to suppressed transcription of the inserted human sequence precluding excessive protamine incorporation during nuclear remodeling.

Prior mouse models in which the human protamine gene cluster integrated as multi-copy insertions also exhibited reduced levels of expression relative to the endogenous protamines^37^, but was expressed independent of its site of integration. In all transgenic lines bearing the 40 kb human sequence transcription of the transgenes was restricted to the testis and conformed to the expected temporal and spatial patterns of expression^14^,^26^. This supports a model in which full expression of the locus cannot be promoted solely from regulatory elements within the inserted sequence despite their ability to insulate against ectopic effects.

The elevated nucleosome coverage observed within the transgenic human protamine locus and the reduced expression levels of the transgenes suggested that locus control was not solely dependent on the integrated sequence but driven by other factors. Recent reports have characterized the impact of higher order chromatin structures on regulating genomic function^46^. Three-dimensional genomic organization is functionally stratified into large sub-chromosomal compartments correlated with either active or silent chromatin^10^. Further partitioning of these compartments identifies regional preferences in chromatin interactions which form the basis of TADs^12^. At this level of genomic organization, interactions between discrete loci occur with increased frequency within domains relative to across domain boundaries^11^. This is best characterized by intra-domain DNA looping events, such as those observed between promoters and their cognate enhancers^47^. Conserved across species and between cell types, TADs are established through the binding of insulator proteins such as Ctcf. Perturbation of Ctcf binding sites within TAD boundaries alters domain structure and looping interactions consequently impacting gene expression^48^. Analysis of available Hi-C datasets demonstrated that the endogenous human and mouse protamine loci are positioned in TADs that include regulatory features absent or depleted from the subdomain harboring the integrated transgene. The native chromatin environment of the endogenous mouse protamine locus is enriched in intrachromosomal contacts, predicted testis enhancers, spermatid Ctcf ChIP-seq peaks, and regulatory factor footprints predictive of prior spermatogenic function (Fig. 4A). In contrast, the transgenic cluster lies within a large repeat-rich TAD depleted of cis regulatory elements. (Fig. 4B).

In mouse testis, the Prm1, Tnp2, and Prm2 transcripts are respectively the 2^nd^, 5^th^, and 6^th^ most abundant RefSeq RNAs (Supplemental Data 1). These levels reflect the need to nearly repackage the entire histone-bound genome with protamine proteins. This magnitude of expression from a single gene cluster is likely achieved through a DNA looping mechanism in which the protamine promoters are brought into contact with one or more neighboring testis enhancer elements. Such an event is likely mediated by the binding of Ctcf upstream of Tnp2 which is bound to this region in mouse round spermatids. The nuclease footprint corresponding to this factor is also observed overlapping this region in mature sperm suggesting that this interaction is maintained throughout spermiogenesis.

In this model, Ctcf coordinates a DNA looping event promoting the interaction of enhancer and promoter regions driving full locus expression once recruited upstream of the protamine locus. This is expected to occur through the binding of Ctcf within the 5′ region of the protamine locus thereby promoting intra-domain chromatin interactions between the members of the gene cluster and *cis* regulatory elements. It is reasonable to expect that mechanisms regulating expression in haploid cells possessing nucleosome-bound genomes parallel those of their diploid somatic counterparts^11^. Experimental manipulation of Ctcf binding sites results in perturbation of Ctcf mediated looping and locus suppression^48^. Binding of Ctcf to the conserved CTCF motif present in the integrated sequence was likely not impeded, as evidenced by the nuclease footprinting observed in mature transgenic sperm. Accordingly this factor would have not been able to coordinate interactions between the transgenes and enhancers and factor binding sites not present within the integrated sequence. Though relevant candidate enhancers await identification in human testis, in lieu of the proposed native regulatory elements, the transgenes may rely upon neighboring DNAs to contribute to locus control. Integrated within a TAD depleted of intrachromosomal interactions, enhancers and regulatory factor footprints the transgenic human protamine domain exhibited reduced levels of transgenic PRM RNAs. The reduced transcriptional permissivity of the transgenes suggests that although the integrated DNA possessed the necessary cis regulatory information to insulate and promote its own expression, this sequence alone was not able to recapitulate full locus expression when removed from its endogenous chromatin environment. While long-range intra- or inter-chromosomal contacts between the transgenic promoters and distant enhancers cannot be excluded, such events are infrequent^11^,^12^. The transcription of this locus, though reduced relative to the endogenous protamines, likely contributes to its maintenance in a nuclease sensitive conformation following nuclear condensation^8^.

It is known that nuclease sensitive sperm DNAs are enriched in Ctcf motifs^7^ and prior MNase-seq analysis of sperm chromatin has identified this protein and its corresponding nuclease footprint in mouse spermatozoa^20^. However, this study did not comprehensively report whether other chromatin proteins might remain bound in sperm. CENTIPEDE analysis of nucleosomal DNA identified a cohort of regulatory factor footprints that remain bound to sperm chromatin (Fig. 5). These results likely provide a record of past chromatin regulatory action. Footprints corresponding to factors predicted to be bound in sperm were significantly enriched within promoters active in testis as well as within corresponding peaks of active histone modifications. This suggested that the binding of these proteins within the chromatin subdomains housing the endogenous and transgenic protamine loci might have contributed to the varied levels of expression (Fig. 6). These regulatory proteins are notable contenders for driving expression of the protamine gene cluster through Ctcf mediated DNA looping by binding upstream of Tnp2. An interesting association was also observed between genomic regions predicted to be active in testis and associated with inferred sites of Rest binding in sperm. The enrichment of the Rest factor within these regions suggests that it may contribute to transcriptional silencing prior to nuclear condensation.

Sites of predicted factor enrichment also included regions expressed in the early embryo. Though not significantly associated with sites of testis transcription, homeobox domain motifs (Fig. 5C) predicted to be bound in sperm exhibited a significant enrichment upstream of rRNA sequences and within the promoters of the olfactory receptor gene family. Zygotic ribosomal RNA transcription has recently been shown to be required for the first cell division in mouse^39^. This process is dependent on the deposition of H3.3 within the paternal pronucleus by the Hira histone chaperone. Sperm chromatin retention of transcription factors within a nucleosome-associated conformation upstream of rRNA sequences may contribute to this process by serving as sites of nucleation for further histone incorporation^1^. As proposed for the rRNA genes, maintaining the promoter regions of the olfactory receptor gene family sequences in association with bound transcription factors flanked by nucleosomes may prime these regions for early use in the embryo^40^. Sites of predicted Ctcf binding in mature sperm were enriched within the promoter regions of genes differently expressed in pronuclei embryos relative to oocytes (Fig. 7). Together these results suggest that sites of predicted factor binding in mouse sperm may prime zygotic chromatin for early utilization. Though the majority of chromatin proteins, including Ctcf, are evicted during the histone-protamine transition the regulatory factors that persist in mature sperm may impact the next generation (Fig. 8C,D). Preferential retention of Ctcf in sperm demarcating higher order chromatin structures suggests the paternal gamete packages DNA in a manner conducive to the inheritance of global genomic organization following fertilization. This could be achieved during nuclear remodeling, by maintaining large stable chromatin domains at the expense of disrupting the subdomains they envelope^11^,^49^,^50^. If perturbed this could alter phenotype^51^ that may have an epigenetic transgenerational effect.

If paternal chromatin organization is transmitted to the oocyte it is likely that sperm genome architecture may reflect species-specific patterns of embryo development (Fig. 9B). In mouse, preimplantation milestones such as embryonic genome activation^52^,^53^,^54^ and compaction^55^,^56^,^57^ occur earlier than that observed in human or bovine embryos^58^. The retention of regulatory factors within murine spermatozoa would be expected to contribute to the accelerated developmental timing of the mouse embryo. For example, nuclease footprinting in mouse spermatozoa demonstrated that proteins, including members of the homeobox family and Ctcf, are likely situated within histone-bound chromatin and enriched within genomic regions that undergo transcription in the zygote or 2-cell embryo^39^,^40^,^59^. Zygotic transcription from the paternal pronucleus exceeds that from the maternal genome supporting a potential role for these sperm borne regulatory factors in activating paternal chromatin^60^. Whether this might be achieved by recruitment of remodeling and/or transcriptional machinery, such as Hira^39^, remains unclear.

Human sperm chromatin footprints comparable to those observed in mouse were not detected in previously published datasets^6^,^44^ nor in the current study. The consistent absence of these footprints across three independently prepared sets of samples suggests that in man, these factors are not present in the mature gamete. Though limited to a single study, MNase-seq analysis of bull sperm produced similar observations (Fig. 9B). In contrast, analysis of previously published mouse sperm MNase-seq samples consistently identifies regulatory factor footprints flanked by well-spaced polynucleosomal arrays (Supplemental Figures 7 and 11). This suggests that the species-specific packaging of paternal chromatin in mice is an independent adaptation required to support the accelerated murine preimplantation development program relative to that of the evolutionarily distant bovine embryo or the more recent common ancestor shared by mice and man^61^.

Species specific patterns of chromatin packaging in the mammalian paternal gamete are likely not primarily sequence-dependent but also driven by other factors. Analysis of the transgenic human protamine locus predicted that motifs contained within the integrated sequence were occupied in mature sperm. This included the conserved CTCF motif positioned between SOCS1 and TNP2. In human sperm this sequence appears unoccupied supporting the view that sequence alone does not dictate mammalian chromatin structure.

Ctcf footprints identified in mouse sperm are enriched within the promoters of differentially expressed pronuclear genes and preferentially localized to boundaries of mouse ESCs TADs. Maintenance of these interactions following sperm nuclear condensation likely primes the murine paternal genome for rapid initiation of regulatory events, potentially including early embryonic transcription and the establishment of higher order chromatin structures. This feature of mouse reproductive biology appears distinct from that of human or bovine and may be an adaptation to the accelerated preimplantation development of this species.

## Materials and Methods

### Sperm chromatin digestion and sequencing library construction

All procedures were carried out with Wayne State University IRB approval. Animal protocols were carried out in accordance with the approved guidelines of Wayne State University Animal Protocol IACUC A 12-01-13. The use of human tissues was approved by the Wayne State University Human Investigation Committee and carried out under Wayne State University Human Investigation Committee IRB Protocol 095701MP2E(5R). All human samples used in this study were obtained after informed consent. Wild-type C57BL/6 mice were purchased from Charles River Laboratories, Inc, Wilmington, MA. Homozygous transgenic mice from transgenic line HP3.1 were bred as described^14^. Mature spermatozoa were isolated from cauda epididymis and vas deferens harvested from individual 6 month old mice on ice into PBS. Following filtration through an 80 micron mesh, the cells were washed twice and resuspended in 1 mL of a PBS solution containing 0.5% Triton X-100. The absence of somatic cells was confirmed by light microscopy. The cells were incubated for 10 minutes on ice with occasional mixing. Ice cold PBS was added to 10 mL and the cells were washed twice prior to counting with a hemocytomoter. Tritonized sperm suspensions were adjusted to 5 × 10^6^ cells/mL in 5 mM CaCl_2_, 10 mM dithiothreitol, buffered with 50 mM Tris-HCl, pH 7.9. The suspension was placed 37 °C for 30 minutes. The sample was diluted with prewarmed reaction buffer to a final concentration of 5 × 10^6^ cells/mL in 5 mM CaCl_2_, 10 mM dithiothreitol, 2 Kunitz unit/mL MNase (New England BioLabs), buffered with 50 mM Tris-HCl, pH 7.9. The sample was digested for 5 minutes and the reactions were stopped with the addition of 0.5 M EDTA pH 8.0 to a final concentration of 20 mM. The cell suspension was then rotated at 4 °C for 30 minutes prior to centrifugation at 20,000 rcf for 10 minutes. Sperm digested with DNA Fragmentation Factor (DFF) were similarly collected and permeabilized. Following washing sperm were resuspended to 5 × 10^6^ cells/mL in 1x NEB1 [10 mM Bis-Tris-Propane-HCl; 10 mM MgCl_2_; 1 mM dithiothreitol; pH 7.0] (New England BioLabs), supplemented with 10 mM dithiothreitol. Following incubation at 37 °C for 30 minutes the sperm suspension was diluted to 2.5 × 10^6^ cells/mL with prewarmed 1x NEB1. The sample was digested for 16 hours at 37 °C following the addition of 10 units Tobacco Etch Virus (TEV) protease and 24 of μl DFF enzyme.

Human sperm nucleosome libraries were prepared as above with the following adjustments. Following liquefaction sperm were washed twice and frozen as dry pellets in liquid nitrogen. Sperm were thawed on ice and washed twice with PBS prior to permeabilization with Triton X-100 and subject to nuclease digestion as above.

Enzymatically released soluble DNAs were recovered from the supernatant to a fresh screw cap tube and digested overnight with proteinase K at 55 °C in the presence of 1% β-mercaptoethanol. The remaining sperm pellets were washed once with PBS and similarly digested by proteinase K alongside tail clips from the sacrificed mice. The DNAs were recovered by phenol–chloroform extraction, precipitated with ethanol and quantified with Picogreen (Invitrogen). Genomic DNA from wild type and transgenic tail clips were digested with MNase and purified as above. Mononucleosome DNA fragments were resolved by gel electrophoresis and extracted from separate agarose plugs with the Qiagen MinElute Gel Extraction kit. Digested control genomic DNAs were gel size selected (~150 bp) and similarly purified. Recovered DNAs were quantified as above and used to construct multiplexed DNA sequencing libraries with the NEBNext ChIP-Seq kit (New England BioLabs). All libraries were subjected to 50 cycles of paired-end sequencing on the Illumina HiSeq 2500 or MiSeq platforms.

### Testis RNA isolation and sequencing library construction

Total RNAs were separately isolated from the decapsulated testes of four transgenic adult mice. Following homogenization in 0.5 ml RLT buffer (Qiagen) supplemented with 1.5% β-mercaptoethanol (Amresco) with a PRO Scientific 200 homogenizer (PROScientific Inc., Oxford, CT), RNAs were extracted as described^62^,^63^. Total RNAs were DNase treated (Turbo DNase, Ambion) and resolved using the 2100 bioanalyzer (Agilent Technologies, Palo Alto, CA, USA). Prepared mouse total testes RNAs^64^ were used to construct individual RNA-seq libraries according to established protocols^63^. Briefly, pre-amplified cDNA libraries were generated from 5 ng of total testis RNA using the Seq-plex system (Sigma) and used to construct sequencing libraries (DNA Ultra-Low, NEB). RNA-seq libraries were subjected to paired-end sequencing on the Illumina Hi-Seq 2500 platform, as above.

### Analysis of sequencing libraries

DNA sequencing libraries were aligned to the mouse genome assembly mm10 with Bowtie (version 2.0.2)^65^, using the following parameters: bowtie2 -X 1000 –very-sensitive. Transgenic samples were additionally aligned to a custom version of the mm10 build containing the inserted human protamine domain at nucleotide position chr19:39397384-39397385^14^. After removing PCR duplicates analysis of aligned sequencing libraries was restricted to properly paired reads with a quality score ≥10. Sequencing fragment midpoints were counted into discrete 500 bp windows genome wide and compared by Spearman’s rank correlation analysis for all samples. Midpoints were similarly counted in 50 bp windows across a 20 kb region center on mouse Prm2, the site of the integration (chr19:39,397,384-39,397,385), and 20,000 randomly selected regions of equal size. In each independent region pairwise Spearman rank correlation coefficients were calculated using a sliding window of 5 regions.

Transgenic testis RNA sequencing libraries were aligned to the custom mm10 genome with Tophat (version 2.0.9)^66^ using the following parameters: tophat2 -r 30 –mate-std-dev 50 –no-coverage-search. Human testes RNA-seq libraries (GSE69434) were similarly aligned to genome build hg19. Sequencing reads uniquely aligned to RefSeq genes were counted with HTSeq^67^ and used to calculate transcripts per million (TPM)^68^.

Published single-end datasets used in the current study were restricted to a similar criterion ignoring the paired requirement. Processing of alignment files, including midpoint and 5′ calculations, genomic intersections, and Jaccard statistics were carried out with the samtools (version 0.1.19)^69^ and bedtools (version v2.19.1–2)^70^ suites. Mappability tracks (50 bp) were constructed using the GemTools suite^71^. ChIP-seq peaks were identified from spermatid datasets^22^ with the MACS2 software suite using default settings^72^. Heatmaps and figures were generated using the ggplot2 and the deepTools packages^73^,^74^. Hi-C interaction maps were retrieved from the Hi-C browser (http://promoter.bx.psu.edu/hi-c/)^11^,^12^.

### Isolation and detection of protamines

Basic protamines were independently isolated from wild type and transgenic cauda epididymal mouse sperm and ejaculated human sperm. All samples contained at least 10 million cells. Spermatozoa were lysed by hypotonic shock and the chromatin solubilized as described^75^. The nucleoproteins were then extracted with HCl 0.5 N at 37 °C for 5 minutes and the precipitated with 20% trichloroacetic acid. Nuclear proteins were visualized in acid-urea polyacrylamide gels as described^76^. Finally, intact nuclear proteins were detected by mass spectrometry using high performance liquid chromatography coupled with electrospray ionization and detection with the Q Exactive MS system (Thermo Fisher Scientific).

### Nuclease footprinting analysis

Sequencing read midpoint coordinates and motif positions were prepared for nuclease footprinting analysis with in-house scripts prior to implementation of the CENTIPEDE algorithm in R^4^. Genomes were scanned to identify sequences that best conformed to known position weight matrix motif (PWM) models for each factor tested^77^ by calculating a PWM value measuring the log_2_ likelihood ratio between the probability that the sequence is generated by the PWM model and the background probability (where each nucleotide generated independently and with equal probability). Motif instances with a PWM value >13 were selected (10,000x over random chance). Motifs instances within a 2 kb window exhibiting a mappability score ≥99% were ranked according to their PWM values and the top quintile used for the primary footprinting analysis. The average conservation score (60way.phastCons60wayPlacental) was calculated for each motif. The average 5-base percent GC dinucleotide frequency immediately up- and downstream of each site, excluding the motif sequence, was also calculated to determine the potential impact of sequence context on binding predictions or as a possible experimental confounder. To infer the binding status of a site the CENTIPEDE algorithm was applied to approximately 2 kb windows centered on each selected motif (motif ± 1 kb). CENTIPEDE uses a negative-multinomial distribution to model the total number of reads but also the spatial pattern of fragment midpoints around the motif instances. The model is specific for each TF motif, and then a posterior probability of binding is reported for each motif instance. Subsequently, a generalized linear model was used to evaluate the predictive value of the three scored variables (PWM, GC%, and phastCons) and the posterior probabilities of those factors:


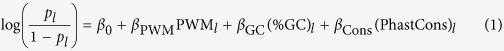


where 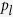 represents the posterior probability of binding at motif instance *l*, PMW_l_ is the PWM value and GC and PhastCons represent the average 5-base percent GC dinucleotide frequency flanking the motif and the conservation score of the motif, respectively. A z-score was calculated for each of the coefficients (PWM value, GC%, and PhastCons score). Factors for which the Z-score associated with the PWM values were greater than or equal to 5 were used for further analysis. Note that the Z-score should not be confused with the values of the dependent variables of the logistic model (1) as it measures by aggregating all the motif locations genome-wide the statistical association between the variables and the footprint locations with high CENTIPEDE posterior values. Within this set of factors motifs exhibiting a posterior probability ≥0.95 were considered bound. The extended analysis of the 5 Mb search regions was performed as described with the following changes. All sites identified within the search regions meeting the following criterion were included in the footprint analysis regardless of their PWM rank: mappability scores ≥90%, PWM values ≥14. A reduced stringency analysis of potential Ctcf sites was also undertaken in which all motifs with a PWM value ≥13 were evaluated. The completed set of occupied Ctcf sites in sperm were used to intersect spermatid ChIP-seq peaks^22^ and promoters of genes differentially expressed in pronuclear embryos^59^. The 5′ genomic start site of sequencing reads was used for single-end sequencing CENTIPEDE analysis.

Footprints were lifted over to Mm 9 genome build for comparisons with ENCODE datasets^36^,^78^. ChIP-seq datasets were obtained from http://chromosome.sdsc.edu/mouse/download/testes.zip. Previously generated enhancer coordinates predicted by a random-forest based algorithm were retrieved from http://promoter.bx.psu.edu/ENCODE/predicted_enhancer_mouse.tar.gz. Peak midpoint files were converted to BED format prior to analysis. ESC Topological Associated Domain (TAD) boundaries were considered to be 4 kb regions centered on the start and end positions of each domain. Significance values for genomic intersections were calculated using a Fisher exact test unless otherwise noted. Promoter regions were considered to span 5 kb upstream and 0.25 kb downstream from transcription start sites (TSSs) with respect to strand orientation. Testis promoter regions corresponded to genes exhibiting non-zero coverage in all testis RNA-seq samples. Pronuclei embryo promoters corresponded to the subset of genes identified as differentially expressed in pronuclear embryos and oocytes^59^.

## Additional Information

**Accession Number**: GEO accession number: GSE78075. http://www.ncbi.nlm.nih.gov/geo/query/acc.cgi.

**How to cite this article**: Johnson, G. D. *et al.* Nuclease Footprints in Sperm Project Past and Future Chromatin Regulatory Events. *Sci. Rep.* **6**, 25864; doi: 10.1038/srep25864 (2016).

## Supplementary Material

Supplementary Information

## Figures and Tables

**Table 1:** Regulatory Factors in Sperm Overlapping Testis Enhancers.

Factor	Footprint Coordinates	Testis Enhancer Peak^1^
Ctcf	chr16: 10,639,310 – 10,641,331	chr16: 10,639,400
Homeobox	chr16: 10,675,153 – 10,677,169	chr16: 10,676,700
Foxj2	chr16: 10,701,314 – 10,703,331	chr16: 10,703,100
Foxj2	chr16: 10,713,448 – 10,715,465	chr16: 10,713,800
Homeobox	chr16: 10,842,275 – 10,844,301	chr16: 10,844,000
Ctcf	chr16: 10,845,809 – 10,847,828	chr16: 10,846,800
Ctcf	chr16: 10,867,176 – 10,869,197	chr16: 10,868,600

^1^(Shen, Yue *et al.* 2012).
