# Supplementary material for: Nuclease Footprints in Sperm Project Past and Future Chromatin Regulatory Events
Source: Sci Rep. 2016 May 17;6:25864. doi: 10.1038/srep25864 (PMC4869110; doi:10.1038/srep25864)

NUCLEASE FOOTPRINTS IN SPERM PROJECT PAST AND FUTURE CHROMATIN REGULATORY EVENTS

SUPPLEMENTAL INFORMATION

(SUPPLEMENTAL TABLE, FIGURE LEGENDS, FIGURES)

Graham D. Johnson[1], Meritxell Jodar[1, 2], Roger Pique-Regi[1, 2] and Stephen A. Krawetz[1, 2*]

[1]Center for Molecular Medicine and Genetics, Wayne State University School of Medicine, Detroit, MI, 48201, USA, [2]Department of Obstetrics and Gynecology, Wayne State University School of Medicine, Detroit, MI, 48201, USA,

[*] To whom correspondence should be addressed. C.S. Mott Center for Human Growth and Development, 275 East Hancock, Detroit, MI, 48201, USA Tel: +1 313 577 6770; Fax: +1 313 577 8554; Email: steve@compbio.med.wayne.edu

Running: SPECIES SPECIFIC RETENTION OF CTCF IN MOUSE SPERM

Supplemental Information

Supplemental Table I. Occupied sperm motifs identified by CENTIPEDE

Supplemental Figure 1. Gene poor regions of the mouse genome are not enriched in nucleosomes.

Supplemental Figure 2. The human protamine chromatin subdomain.

Supplemental Figure 3. Chromatin interaction data from available cell types

Supplemental Figure 4. CENTIPEDE binding predictions were correlated across all sperm samples.

Supplemental Figure 5. Hierarchical clustering identifies binding at identical homeobox motifs.

Supplemental Figure 6. Homeobox motifs.

Supplemental Figure 7. The sperm Ctcf footprint is observed in modified and variant histone sequencing data.

Supplemental Figure 8. Boris and Ctcf associations in sperm are retained from round spermatids.

Supplemental Figure 9. Distances between Ctcf footprints in sperm.

Supplemental Figure 10. CTCF footprints are not observed in human sperm.

Supplemental Figure 11. Detection of mouse sperm Ctcf footprints is not protocol dependent.

Supplemental Figure Legends

Supplemental Figure 1. Gene poor regions of the mouse genome are not enriched in nucleosomes. (A) Mononucleosome sequencings fragment midpoints are plotted against gene density as in Carone, B.R., et al. (B) Mononucleosomes are enriched proximal to transcription start sites (TSS). The $\log_2$ ratio of library size normalized mononucleosome and digested control DNA paired-end sequencing midpoints are presented within 10 kb windows centered on TSSs for all RefSeq genes. (C) MNase digestion of mouse sperm chromatin was not driven to mononucleosomes. Left lane, ladder; middle lane, MNase-released soluble DNAs; right lane, insoluble pellet.

Supplemental Figure 2. The human protamine chromatin subdomain. The approximately 40 kb human protamine sequence inserted in mouse corresponds to less than 10% of the contour length contained within the chromatin interaction domain housing these genes in their native state (dashed box). Human Hi-C interaction data is from GM12878 [1]. The locations of the endogenous protamine domains are highlighted in yellow. The site of transgene insertion within mouse chromosomes 19 is marked on a separate track. Peaks of Hi-C interaction frequencies containing the loci of interest are demarcated by dashed lines and are considered chromatin subdomains.

Supplemental Figure 3. Chromatin interaction data from available cell types. Mouse Hi-C cortex (left) and ESCs (right) data corresponding to either the 5 Mb search region centered on the endogenous mouse protamine locus (A) or the insertions site (B). Data generated in cortex and ESCs are at lower resolution than CH-12 cells [Figure 4; 2]. (C) Alternative Hi-C data for the 5 Mb region centered on the human protamine locus. Six additional datasets are available and all are of the same resolution as the primary figure [1]. The location of the protamine domain is highlighted in yellow and bracketed by dotted lines demarcating its position within the chromatin subdomains.

Supplemental Figure 4. CENTIPEDE binding predictions were correlated across all sperm samples. (A) Density plots of pairwise posterior probability correlations for all individual samples for each factor in the union of factors exhibiting a PWM Z-score ≥ 5 in sperm. The posterior probability of all sites were used for Spearman rank correlation analysis. (B) Sperm nucleosome data was pooled and reanalyzed with CENTIPEDE. As in A, correlations were performed with posterior probabilities derived from pooled sperm nucleosome data (n = 3) and digested naked DNAs.

Supplemental Figure 5. Hierarchical clustering identifies binding at identical homeobox motifs. Two groups are identified by hierarchical clustering on genomic distributions of bound factor sites (Jaccard index). The homeobox factors (purple) share a common motif resulting in overlapping predictions of binding. Asterisks denote motifs identified in other species with no known mouse homolog.

Supplemental Figure 6. Homeobox motifs. Motifs corresponding to the factors in the second cluster (Figure 5, dashed box; Supplemental Figure 3; purple box).

Supplemental Figure 7. The sperm Ctcf footprint is observed in modified and variant histone sequencing data. Ctcf footprints were identified in MNase ChIP-seq datasets from mature mouse sperm with CENTIPEDE [3]. The use of single-end sequencing data required plotting the 5' ends of sequencing reads.

Supplemental Figure 8. Boris and Ctcf associations in sperm are retained from round spermatids. (A) A subset of Ctcf motifs bound in sperm lacked a corresponding Ctcf ChIP-seq peak in round spermatids [n = 1,857, 23.3% of sperm Ctcf footprints; 4]. This was due to the ChIP-seq signal failing to reach significance and not the presence of exclusive binding within the gamete. (B) Heatmaps of relative sequencing coverage in 2 kb windows centered on the complete set of 7,967 Ctcf sites predicted to be bound in mature mouse sperm. Data are

presented for ChIP-seq against Boris in round spermatids, sperm nuclease-seq, and digested naked DNA libraries (left, center, and right, respectively). Sites are presented in descending order of coverage within the Boris ChIP-seq samples [4].

Supplemental Figure 9. Distances between Ctcf footprints in sperm. The distribution of intra-chromosomal lengths between occupied Ctcf footprints in sperm is presented. Sites are separated by a median distance of 0.23 Mb on average.

Supplemental Figure 10. CTCF footprints are not observed in human sperm.

Supplemental Figure 11. Detection of mouse sperm Ctcf footprints is not protocol dependent. CENTIPEDE analysis showed that the majority of Ctcf footprints identified in the current study were also present in a previously published sperm mononucleosome dataset[5].

Supplemental Table I

Supplemental Table I. Occupied sperm motifs identified by CENTIPEDE

MOTIF ID	DATABASE GENE NAME	MOUSE GENE SYMBOL	MOUSE GENE NAME	SPEARMAN RHO*	NUCLEOSOME PERIODICITY	HOMEOBOX MOTIF	AVERAGE TESTIS TPM	TESTIS TPM STDEV	SPECIES OF OBSERVED MOTIF[†]
M01432	HOXD8	Hoxd8	Homeobox D8	0.21	TRUE	TRUE	25.43	1.21	
M01200	CTCF	Ctcf	CCCTC-binding factor	-0.03	TRUE	FALSE	24.21	1.75	
M01259	CTCF	Ctcf	CCCTC-binding factor	-0.03	TRUE	FALSE	24.21	1.75	
MA0139.1	CTCF	Ctcf	CCCTC-binding factor	-0.10	TRUE	FALSE	24.21	1.75	
M01474	Esx1	Esx1	ESX homeobox 1	0.23	TRUE	TRUE	21.04	7.57	
MA0138.2	REST	Rest	RE1-silencing transcription factor	0.12	TRUE	FALSE	18.34	2.42	
M00138	Oct1	Pou2F1	POU domain, class 2, transcription factor 1	0.26	TRUE	FALSE	13.00	0.93	
M01354	Oct1	Pou2F1	POU domain, class 2, transcription factor 1	0.21	TRUE	TRUE	13.00	0.93	
M00422	FOXJ2	Foxj2	Forkhead box j2	0.29	TRUE	FALSE	4.62	0.58	
M01348	K-2b	Prrx1	Paired related Homeobox 1	0.25	TRUE	TRUE	4.52	0.77	
M01406	HOXC6	Hoxc6	Homeobox C6	0.21	TRUE	TRUE	1.77	0.52	
M01367	Lhx9	Lhx9	LIM Homeobox protein 9	0.22	TRUE	TRUE	1.57	0.44	
M01328	Isl2	Isl2	Insulin related protein 2	0.24	TRUE	TRUE	1.08	0.48	
M01420	Ncx	Tlx2	T cell leukemia, homeobox 2	0.22	TRUE	TRUE	1.01	0.39	
M01391	pax6	Pax6	Paired box 6	0.24	TRUE	TRUE	0.72	0.21	
M01316	Oct_6	Pou3F1	POU domain, class 3, transcription factor 1	0.23	TRUE	TRUE	0.64	0.19	
M01454	HOXC5	Hoxc5	Homeobox C5	0.23	TRUE	TRUE	0.61	0.19	
M01477	Octamer	Pou3F2	POU domain, class 3, transcription factor 2	0.23	TRUE	TRUE	0.48	0.17	
M01353	Lhx5	Lhx5	LIM homeobox protein 5	0.23	TRUE	TRUE	0.40	0.24	
M01355	ALX-3	Alx3	Aristaless-like Homeobox 3	0.24	TRUE	TRUE	0.10	0.12	
M01431	Barx-2	Barx2	BarH-like Homeobox 2	0.22	TRUE	TRUE	0.10	0.20	
M01356	PMX2B	Phox2B	Paired-like homeobox 2b	0.23	TRUE	TRUE	0.10	0.13	
M00510	Lhx3a	Lhx3A	LIM homeobox protein 3	0.22	TRUE	TRUE	0.08	0.10	
M01471	Lhx3	Lhx3	LIM homeobox protein 3	0.23	TRUE	TRUE	0.08	0.10	
M01971	LHX3b	Lhx3B	LIM homeobox protein 3	0.24	TRUE	TRUE	0.08	0.10	
MA0135.1	Lhx3	Lhx3	LIM homeobox protein 3	0.22	TRUE	TRUE	0.08	0.10	
M01360	Dbx-2	Dbx2	Developing Brain Homeobox 2	0.22	TRUE	TRUE	0.08	0.09	
M01424	HOXB4	Hoxb4	Homeobox B4	0.23	TRUE	TRUE	0.03	0.07	
M00724	HNF3alpha	Foxa3	Forkhead box a3	0.37	TRUE	FALSE	0.03	0.06	
M01335	Vsx1	Vsx1	Visual System Homeobox 1 homolog	0.23	TRUE	TRUE	0.03	0.05	
M00791	HNF3	Foxa2	Forkhead box a	0.32	TRUE	FALSE	0.00	0.00	
M01363	lmx1b	Lmx1B	LIM homeobox transcription factor 1	0.23	TRUE	TRUE	0.00	0.00	
M01465	Pit-1	Pou1F1	POU domain, class 1, transcription factor 1	0.22	TRUE	TRUE	0.00	0.00	
M01473	Brn-4	Pou3F4	POU domain, class 3, transcription factor 4	0.21	TRUE	TRUE	0.00	0.00	
M01483	Dbx-1	Dbx1	Developing Brain Homeobox 1	0.26	TRUE	TRUE	0.00	0.00	
M00999	AIRE	Aire	Autoimmune regulator	0.29	FALSE	FALSE	0.99	0.63	
M01004	Helios	Ikzf2	IKAROS Family zinc finger 2	0.26	FALSE	FALSE	0.30	0.07	
M01261	HNF3A	Foxa1	Forkhead box a1	0.20	FALSE	FALSE	0.03	0.06	
M00734	CIZ	Zfp384	Zinc Finger protein 384	0.36	FALSE	FALSE	27.01	1.30	
M00030	MATa1			0.31	FALSE	FALSE			Saccharomyces Cerevisiae
M00165	HSF			0.19	FALSE	FALSE			Drosophila
M00169	HSF			0.23	FALSE	FALSE			Saccharomyces Cerevisiae
M00343	RAV1			0.13	FALSE	FALSE			Arabidopsis Thaliana
M00503	ATHB-5			0.26	FALSE	FALSE			Arabidopsis Thaliana
M01504	Nhp6b			0.22	TRUE	FALSE			Saccharomyces Cerevisiae
M01528	SFP1			0.29	FALSE	FALSE			Saccharomyces Cerevisiae
M01566	Sfl1			0.27	FALSE	FALSE			Saccharomyces Cerevisiae
M01827	GT-1			0.32	FALSE	FALSE			Arabidopsis Thaliana
MA0149.1	EWSR1-FLI1			0.23	TRUE	FALSE			Human
PBM0036	GR09/Sfl1			0.27	FALSE	FALSE			Saccharomyces Cerevisiae
PBM0039	GR09/Sfp1			0.29	FALSE	FALSE			Saccharomyces Cerevisiae
PBM0051	GR09/Nhp6b			0.21	TRUE	FALSE			Saccharomyces Cerevisiae

*Merged mononucleosome libraries and controls

†Factor not described in mouse

Supplemental References

1. Rao, S.S., et al., *A 3D map of the human genome at kilobase resolution reveals principles of chromatin looping.* Cell, 2014. **159**(7): p. 1665-80.
2. Dixon, J.R., et al., *Topological domains in mammalian genomes identified by analysis of chromatin interactions.* Nature, 2012. **485**(7398): p. 376-80.
3. Erkek, S., et al., *Molecular determinants of nucleosome retention at CpG-rich sequences in mouse spermatozoa.* Nat Struct Mol Biol, 2013. **20**(7): p. 868-75.
4. Pugacheva, E.M., et al., *Comparative analyses of CTCF and BORIS occupancies uncover two distinct classes of CTCF binding genomic regions.* Genome Biol, 2015. **16**(1): p. 161.
5. Carone, B.R., et al., *High-resolution mapping of chromatin packaging in mouse embryonic stem cells and sperm.* Dev Cell, 2014. **30**(1): p. 11-22.

Supplemental Figure 1

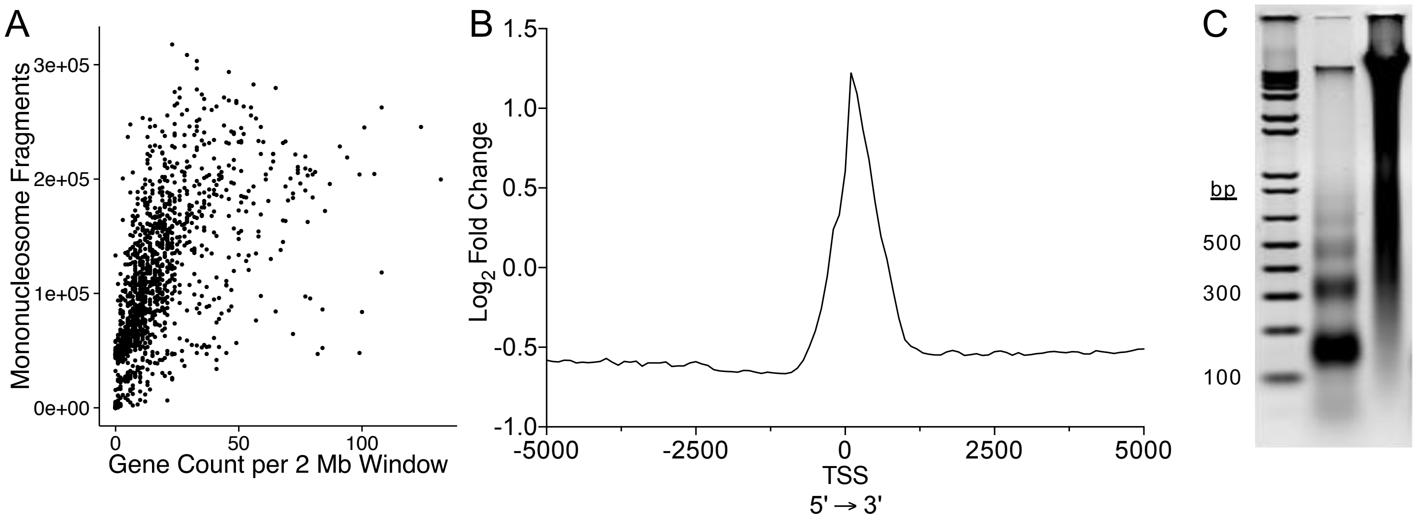

Supplemental Figure 2

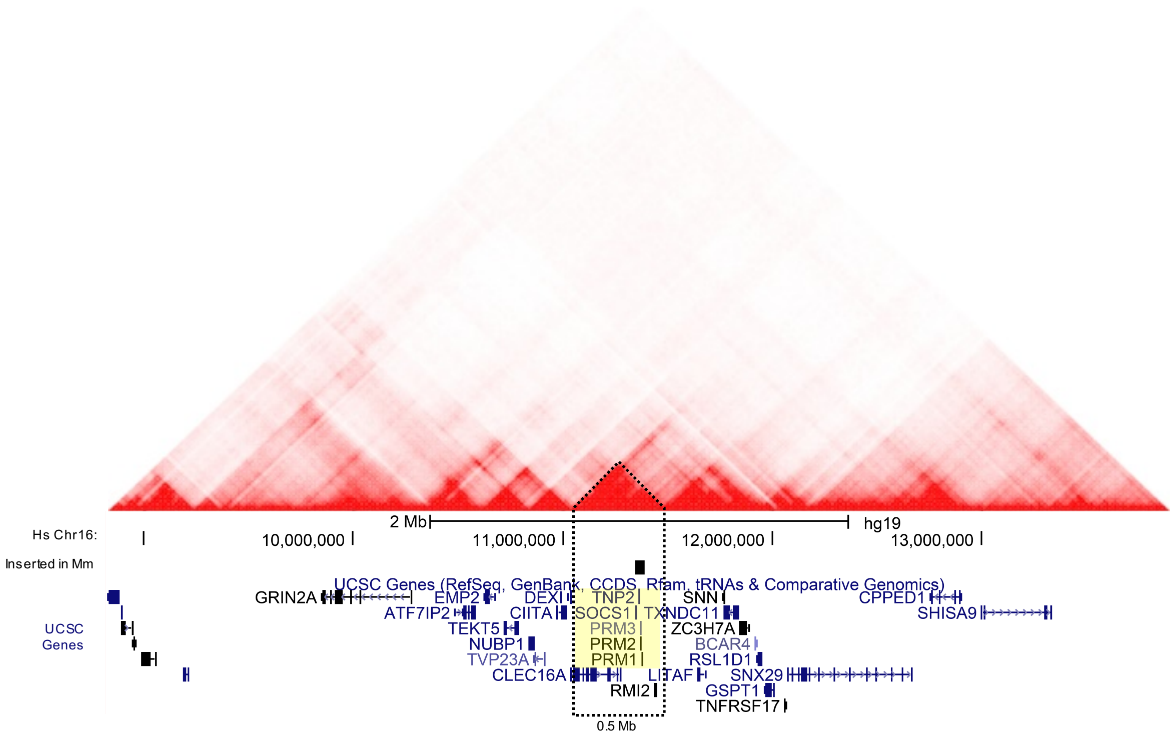

Supplemental Figure 3A, B

A

Mouse protamine locus

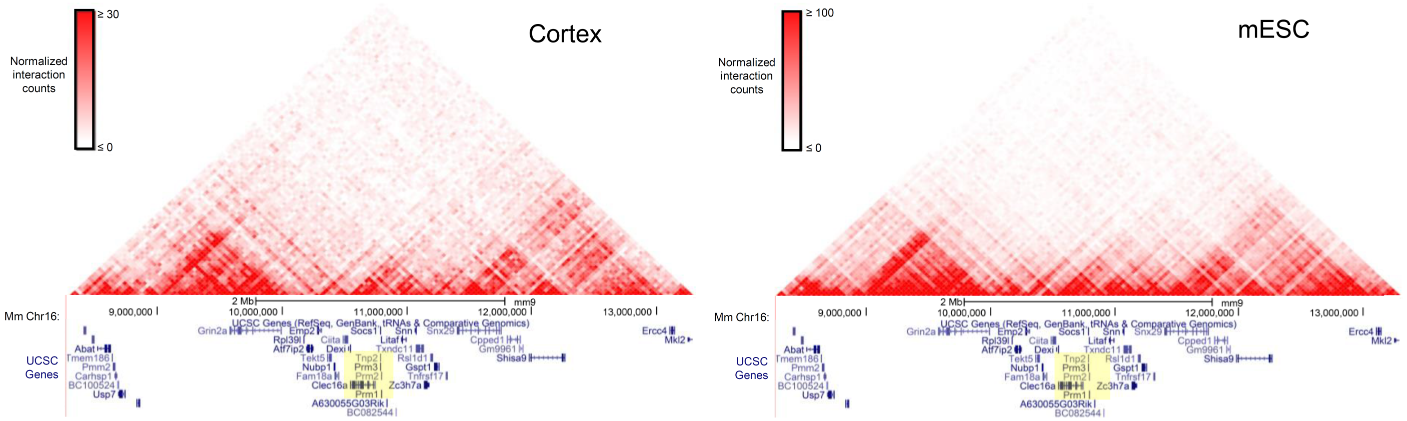

B

Insertion site

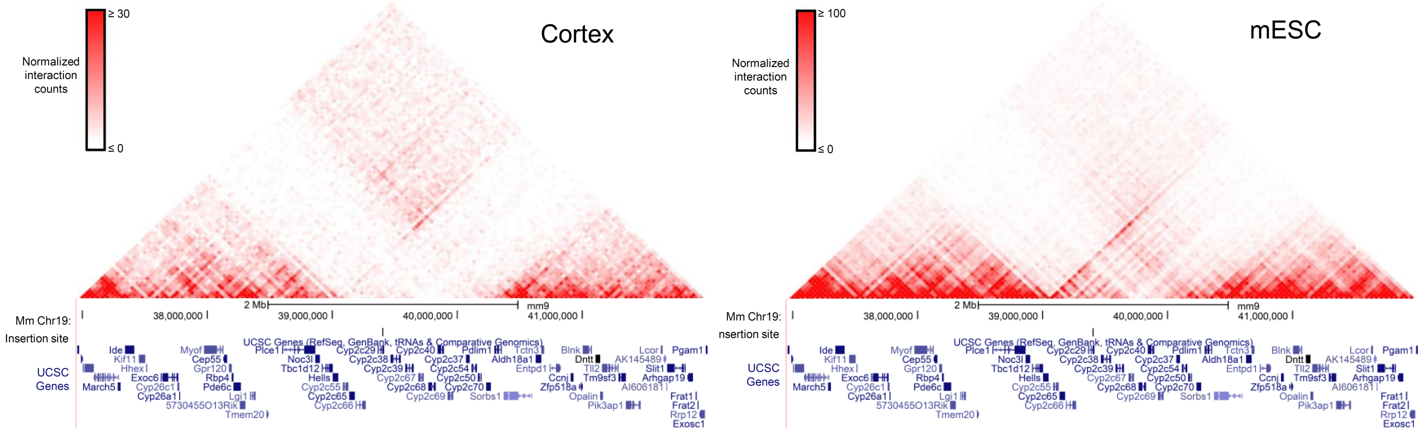

Supplemental Figure 3C

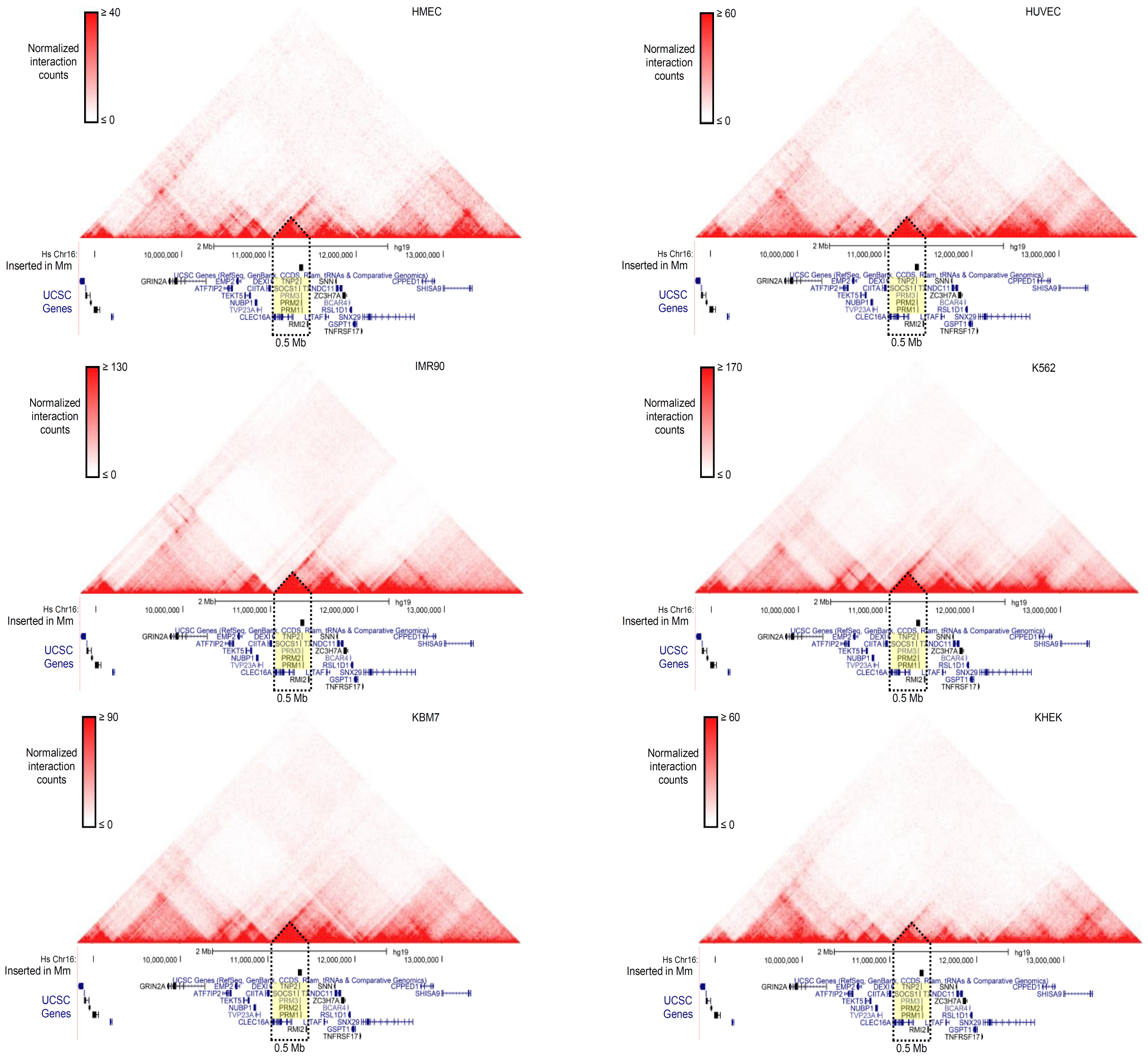

Supplemental Figure 4

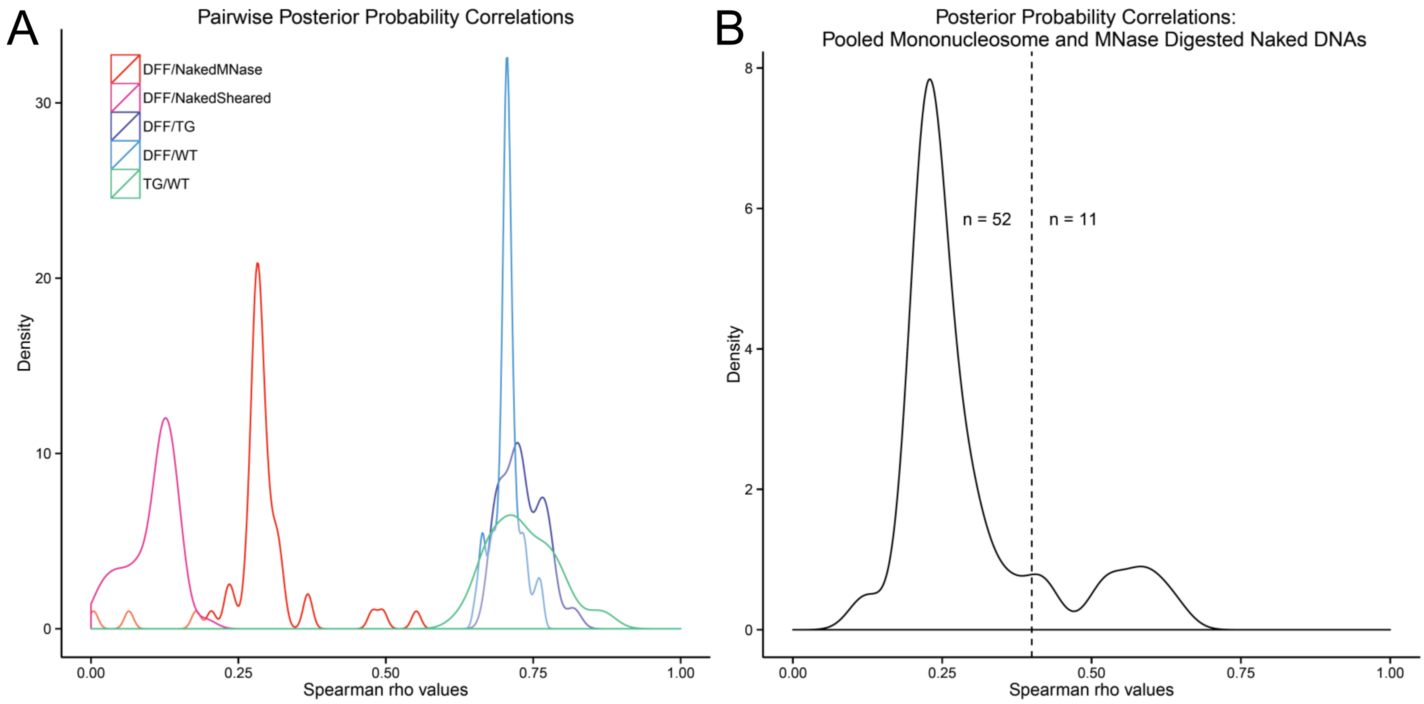

Supplemental Figure 5

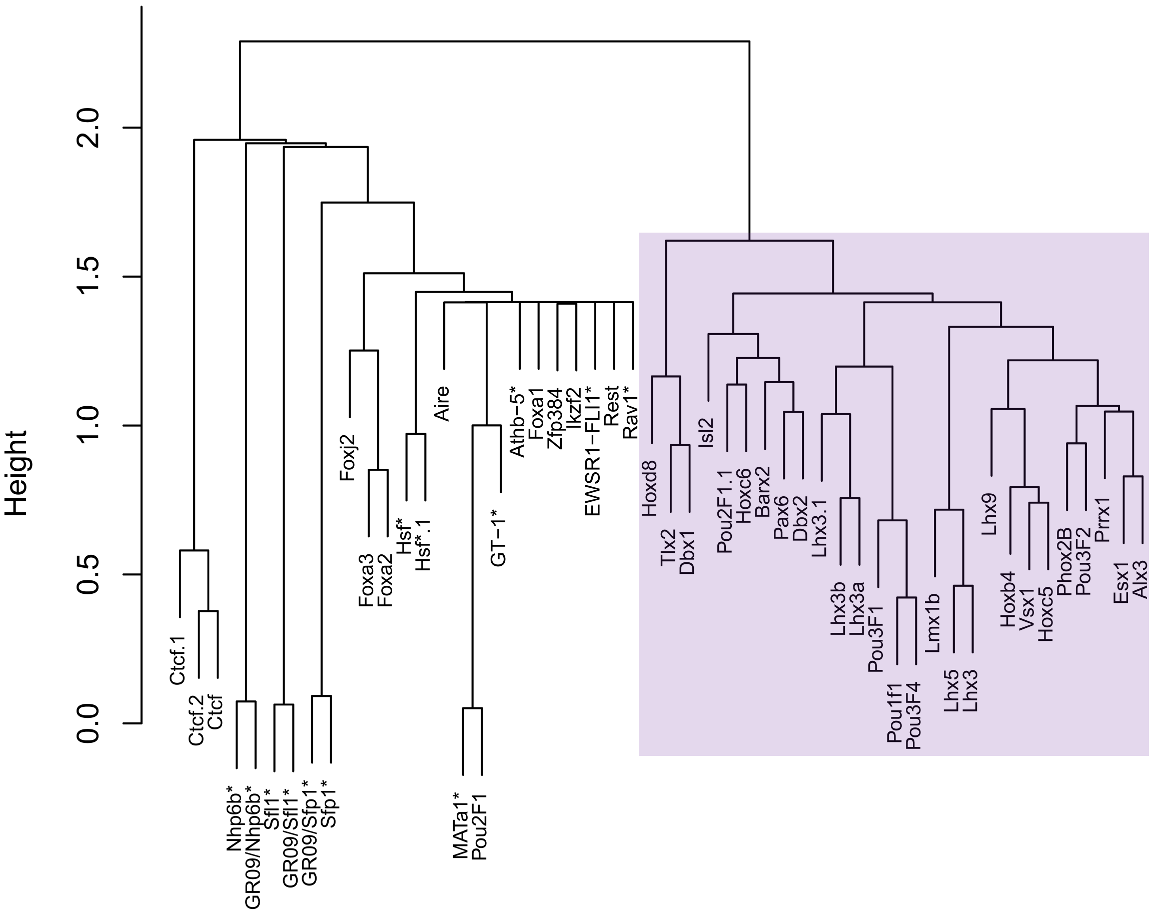

Supplemental Figure 6

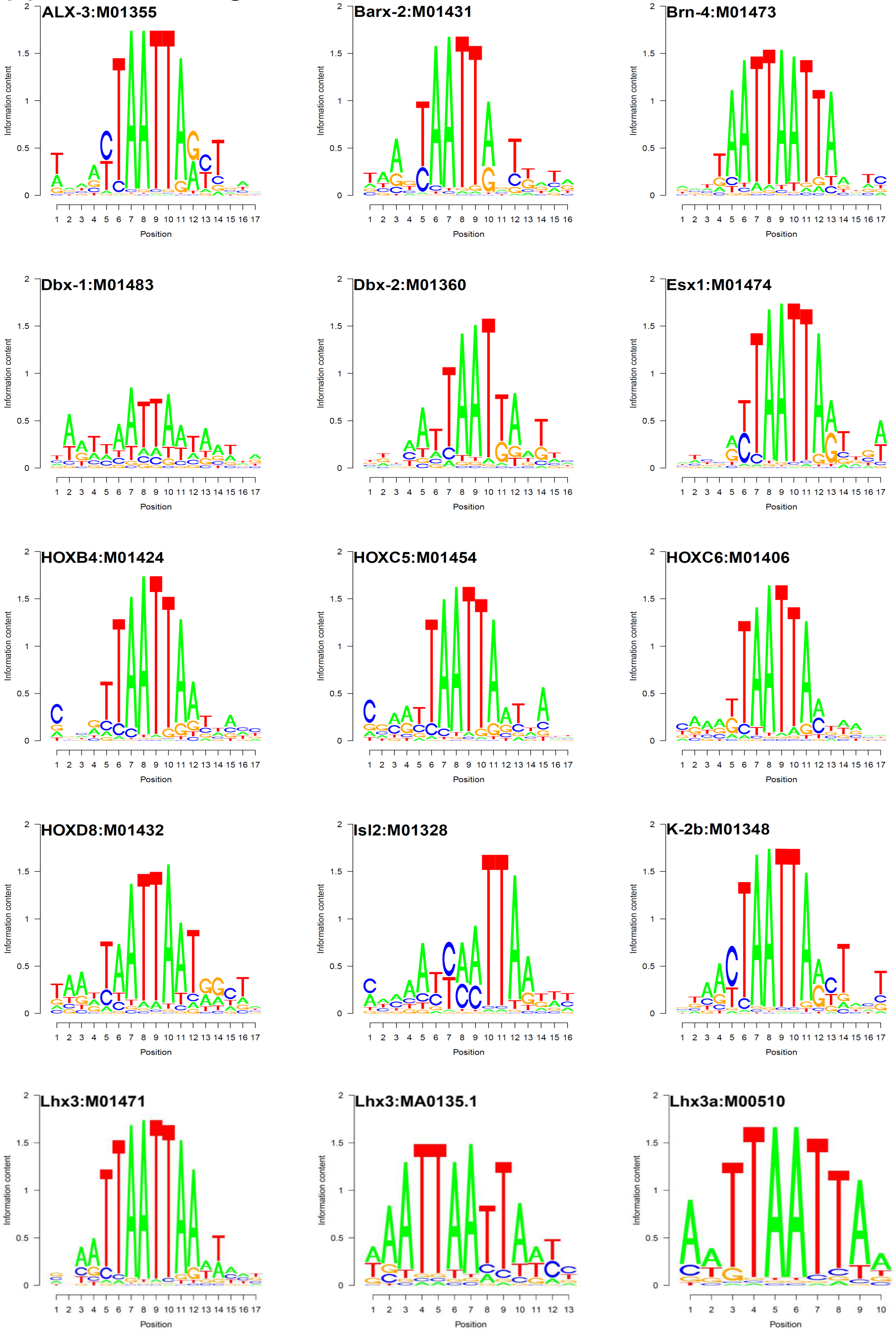

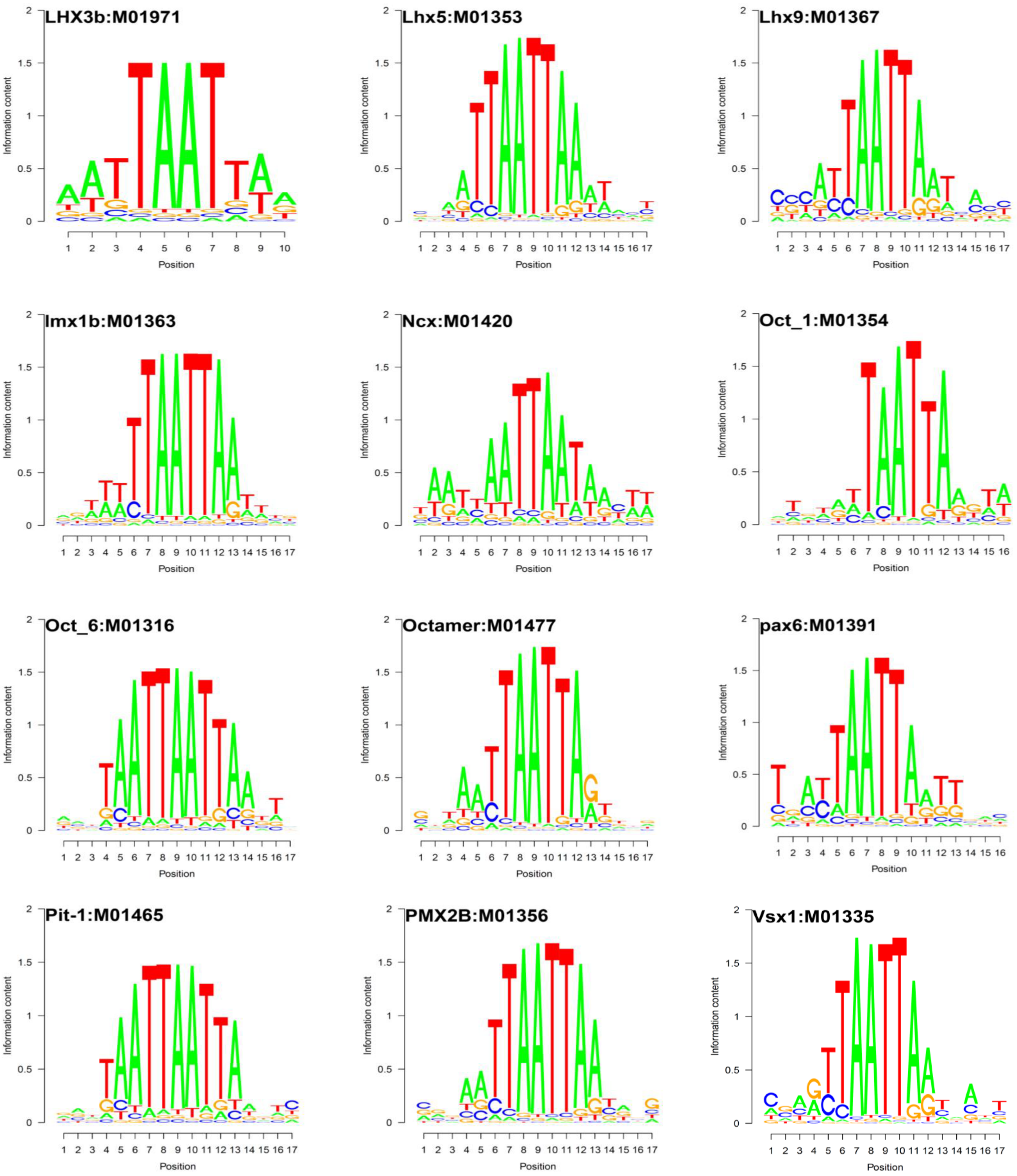
LHX3b:M01971
Lhx5:M01353
Lhx9:M01367
lmx1b:M01363
Ncx:M01420
Oct_1:M01354
Oct_6:M01316
Octamer:M01477
pax6:M01391
Pit-1:M01465
PMX2B:M01356
Vsx1:M01335
Information content
Position

Supplemental Figure 7

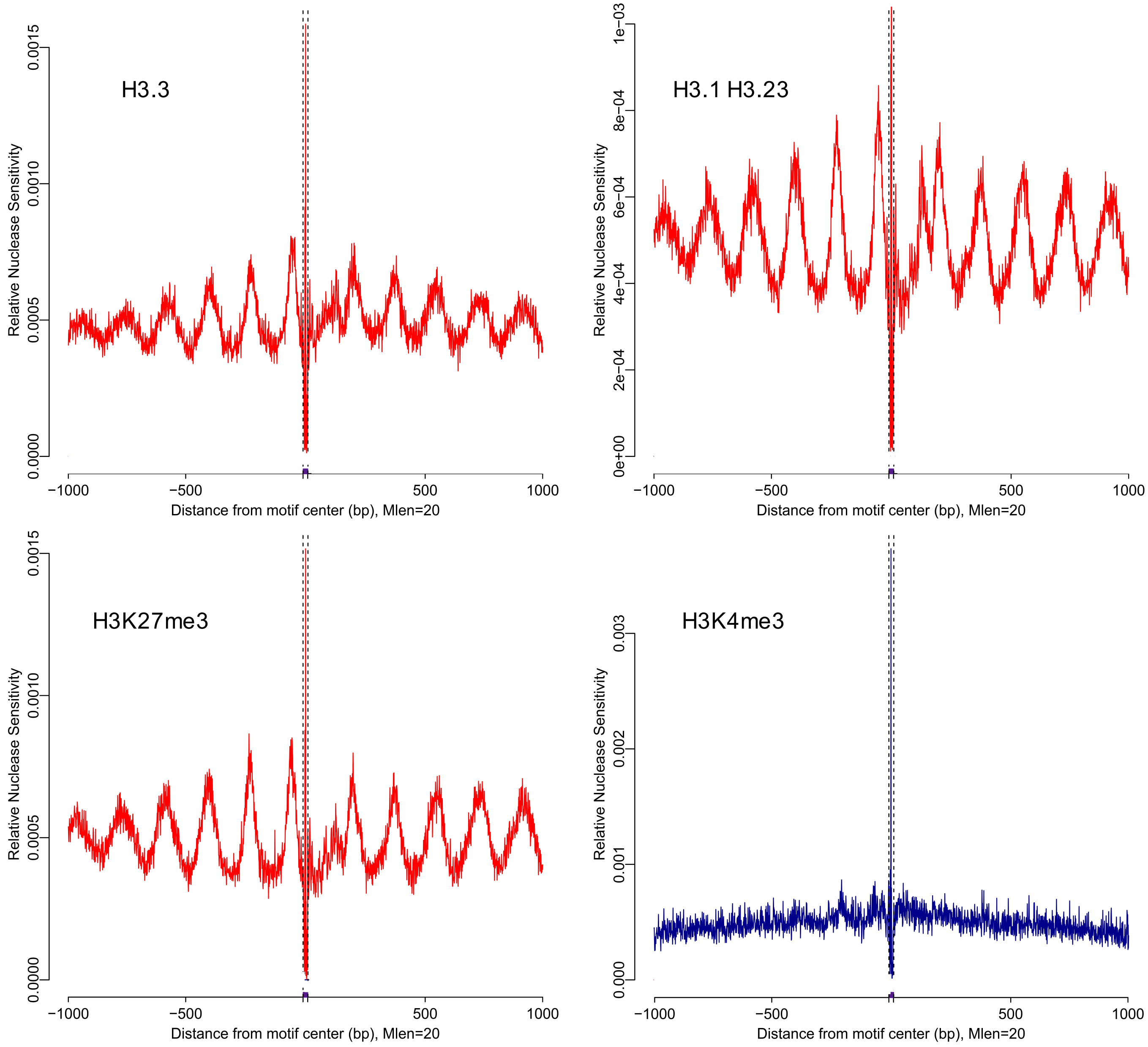

Supplemental Figure 8

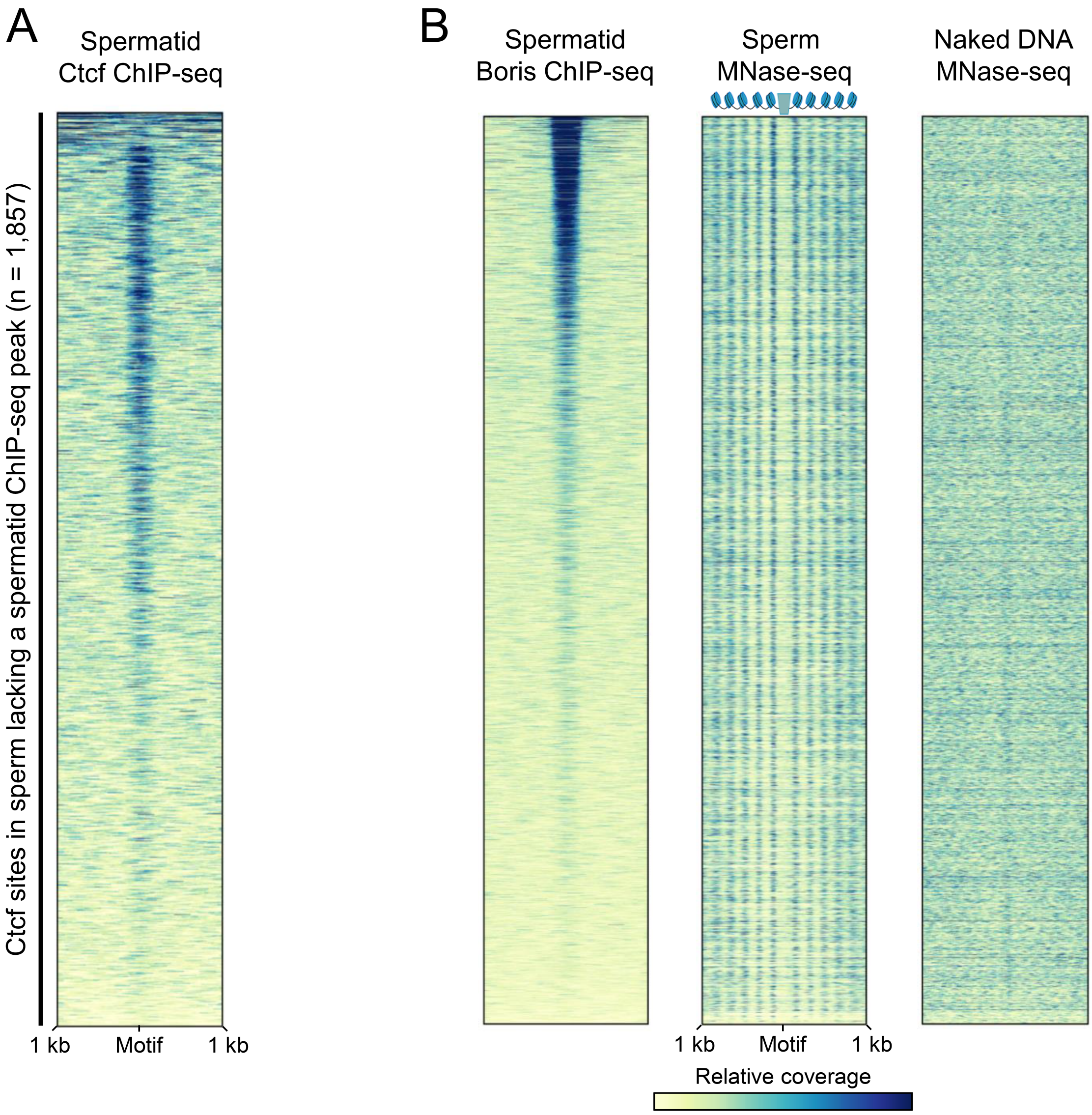

Supplemental Figure 9

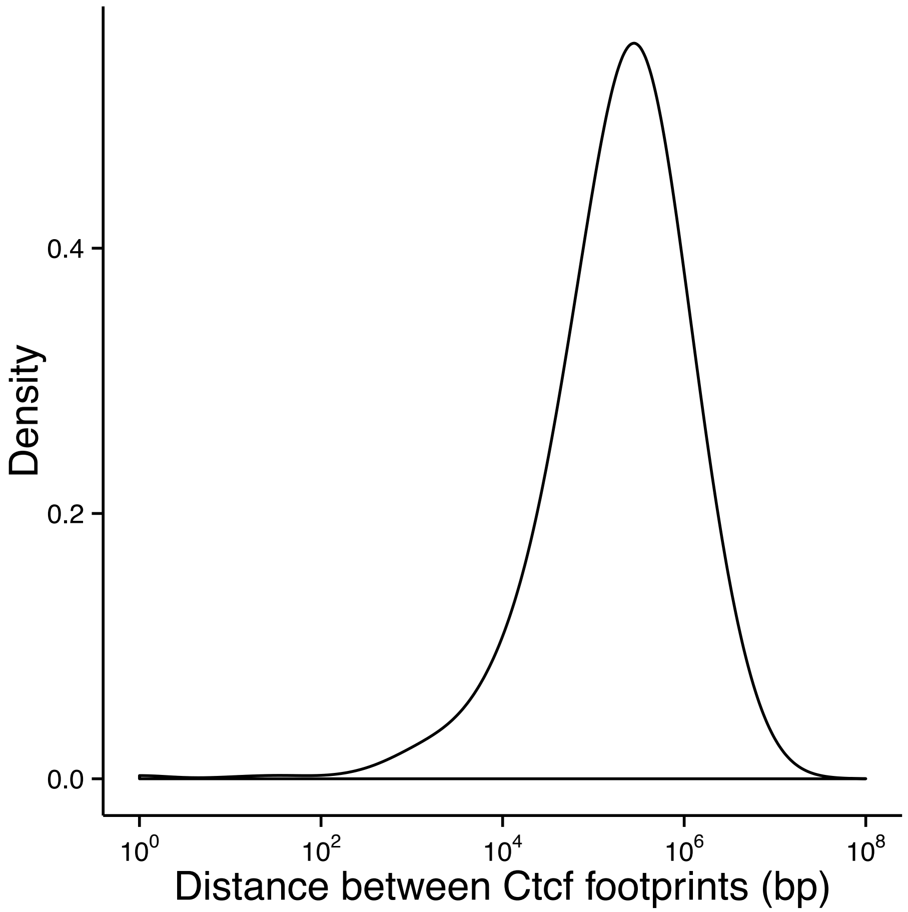

Supplemental Figure 10

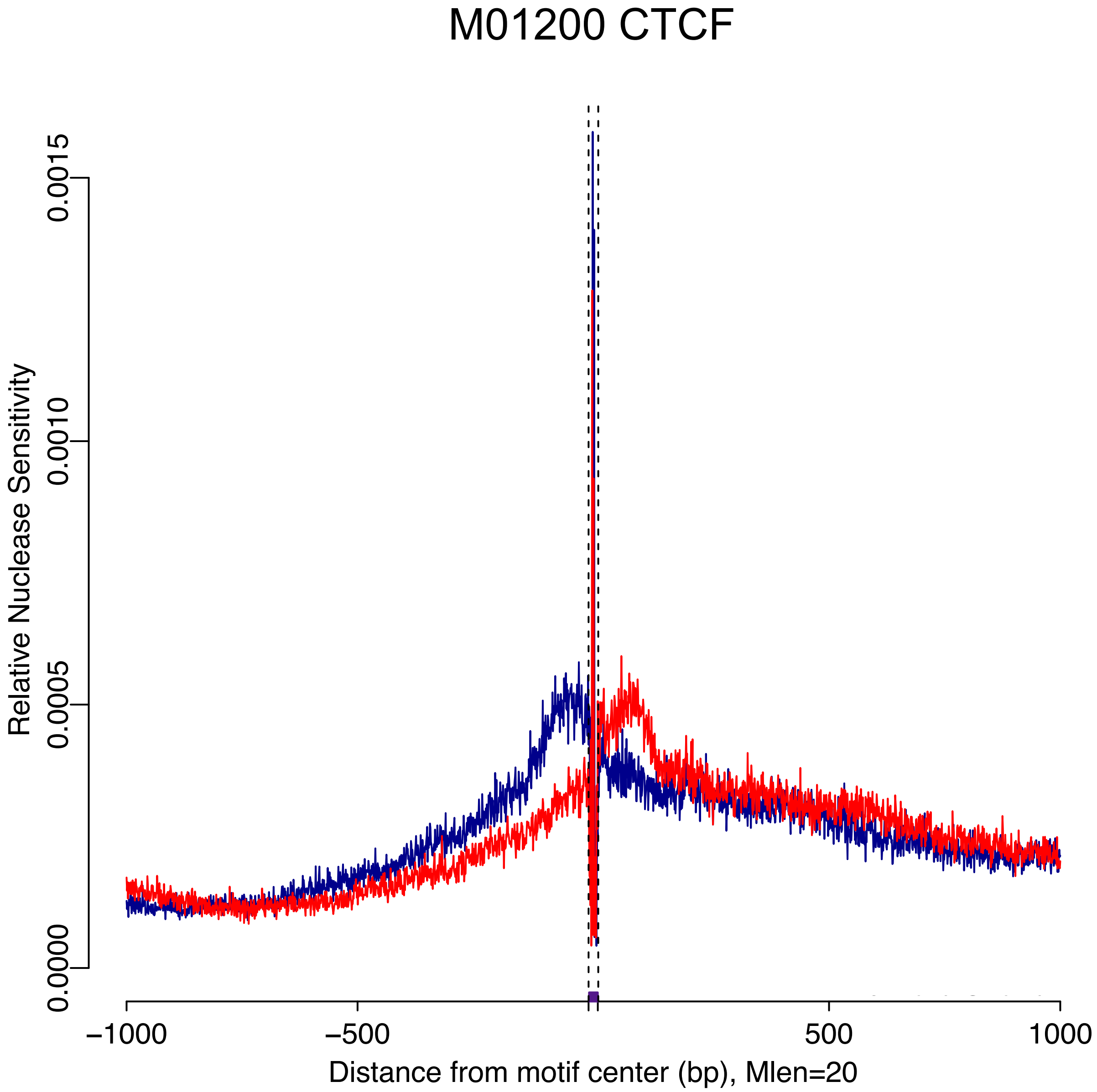

Supplemental Figure 11

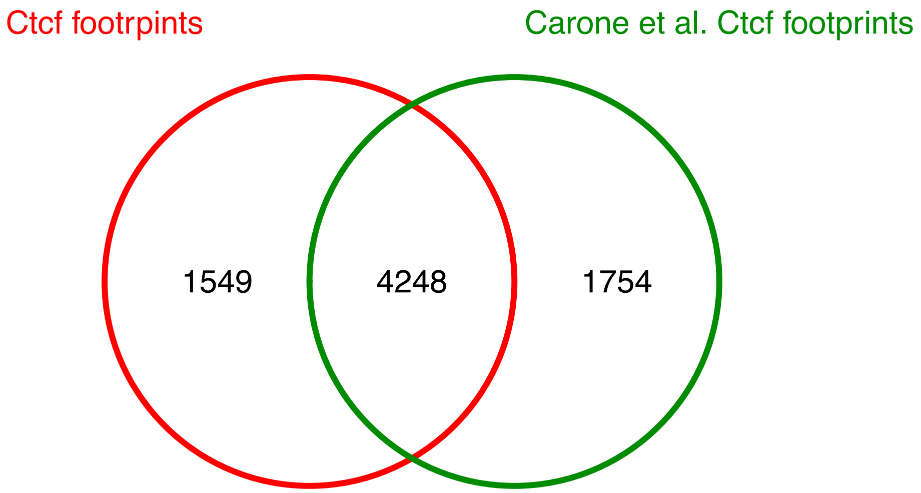

Supplement: Supplementary Information [file srep25864-s1.pdf]
